# The Efficacy and Safety of Plozasiran on Lipid Profile in Dyslipidemic Disorders: A Systematic Review and Meta-Analysis

**DOI:** 10.1007/s10557-025-07798-8

**Published:** 2025-11-18

**Authors:** Pishoy Sydhom, Bakr Al-Quraishi, Aya Gohar, Mohamad El-Shawaf, Nahla Shehata, Miral Ataya, Mark Sydhom, Nouran Awwad, Haidy Motawade, Nourhan Naji, Mahmoud Shaaban Abdelgalil

**Affiliations:** Faculty of Medicine, Ain Shams University, 56th Abbaseyia Street, Cairo, Egypt

## Abstract

**Purpose:**

Emerging lipid-lowering therapies, such as Plozasiran, target apolipoprotein C-III (APOC-III) by inhibiting its hepatic production at the mRNA level, presenting a novel approach to lipid regulation. However, the safety and efficacy of plozasiran have yet to be fully established.

**Methods:**

We searched PubMed, Scopus, Web of Science, and Cochrane CENTRAL register of trials for studies comparing plozasiran to placebo in patients with dyslipidemic disorders. The primary outcomes were percentage changes from baseline in triglyceride (TG) and APOC-III levels at 24 weeks and the end of the study. Secondary outcomes included changes in other lipid parameters and safety outcomes at 24 weeks and the end of the study. A protocol was registered to PROSPERO under registration number [CRD420251026605].

**Results:**

Four studies encompassing 1,514 participants were included in our meta-analysis. Plozasiran significantly improved TGs, APOC-III, non-high-density lipoprotein cholesterol (non-HDL-C), high-density lipoprotein cholesterol (HDL-C), and apolipoprotein B (ApoB) levels at both 24 weeks and study completion. Subgroup analyses based on dose and regimen revealed consistent findings. Quarterly administration of plozasiran at 10 mg, 25 mg, and 50 mg resulted in significant reductions in TGs, APOC-III, non–HDL-C, and HDL-C at both 24 weeks and study completion. For ApoB, all three doses produced significant reductions at 24 weeks; however, only the 25 mg and 50 mg quarterly regimens sustained these reductions through the end of the study. Regarding safety, patients receiving plozasiran experienced a higher incidence of any adverse events, headache, and mild rises in HbA1C levels. Subgroup analysis revealed a dose-dependent pattern for certain safety outcomes.

**Conclusion:**

While Plozasiran shows strong potential as a therapeutic option for severe dyslipidemic conditions, further studies are needed to compare its efficacy and safety with currently available treatments and, more importantly, evaluate its impact on clinical outcomes for implementation in clinical practice.

**Supplementary Information:**

The online version contains supplementary material available at 10.1007/s10557-025-07798-8.

## Introduction

Dyslipidemias are common metabolic disorders characterized by abnormalities in various lipid parameters. They can be broadly classified into primary and secondary forms [1]. Primary dyslipidemias are genetic in origin and include conditions such as familial hypercholesterolemia and familial combined hyperlipidemia. In contrast, secondary dyslipidemias often occur in the context of metabolic syndrome and are commonly associated with obesity, alcohol intake, sedentary lifestyle, and type 2 diabetes [1, 2]. Over recent decades, the prevalence of hypertriglyceridemia has risen to levels comparable to those of type 2 diabetes and obesity [3], particularly affecting one-third of adult men and one-fifth of women, with risk increasing with age [4].

Among genetic forms, familial chylomicronemia syndrome (FCS) is particularly severe, caused by loss-of-function mutations in the lipoprotein lipase (LPL) gene, leading to triglyceride levels often exceeding 880 mg/dL [5, 6]. The absence of LPL activity renders standard lipid-lowering therapies—such as fibrates and omega-3 fatty acids—largely ineffective. Similarly, statins often fail to achieve adequate TG reduction in these patients. Moreover, currently available therapies haven’t been effective enough to reach guideline-recommended therapeutic goals in acquired severe hypertriglyceridemia [5, 7, 8].

All forms of dyslipidemia significantly increase the risk of atherosclerotic cardiovascular disease (ASCVD) and all-cause mortality, primarily due to elevated LDL-C levels, triglycerides (TGs), triglyceride-rich lipoproteins (TRLs), and their remnants [7]. A fasting TG level above 150 mg/dL (1.69 mmol/L) is widely recognized as a marker of increased ASCVD risk [9, 10]. Furthermore, severe hypertriglyceridemia (≥ 500 mg/dL) is associated with serious non-cardiovascular complications, including non-alcoholic fatty liver disease, steatohepatitis, and acute pancreatitis [11, 12].

This therapeutic gap has prompted the search for novel targets that can produce meaningful reductions in TG and TRL levels. One such target is apolipoprotein C-III (APOC-III), a glycoprotein synthesized in hepatocytes and found in several lipoproteins, including chylomicrons (CMs), very-low-density lipoprotein (VLDL), low-density lipoprotein (LDL), and high-density lipoprotein (HDL). It significantly influences lipoprotein metabolism through both lipoprotein lipase (LPL)–dependent and LPL-independent mechanisms. Several APOC-III–directed therapies have been developed, including antisense oligonucleotides (e.g., volanesorsen), small interfering RNA (siRNA) molecules (e.g., plozasiran), and monoclonal antibodies [13, 14]. Plozasiran, a siRNA-based therapeutic, reduces hepatic APOC-III production by inhibiting mRNA transcription [15].

Despite its promising therapeutic potential [16, 17], the efficacy and safety of plozasiran have not yet been comprehensively evaluated through a systematic review or meta-analysis. This study aims to address that gap by analyzing data from randomized controlled trials (RCTs) of plozasiran, with a focus on its impact on lipid parameters and the incidence of adverse events.

## Methods

### Methods and Materials

This systematic review and meta-analysis were performed according to the Preferred Reporting Guidelines for Systematic Reviews and Meta-Analyses (PRISMA) 2020 [18] and adhered to the Cochrane Handbook of Systematic Reviews of Interventions [19]. The protocol was prospectively registered with the International Prospective Register of Systematic Reviews (PROSPERO) under the registration number [CRD420251026605].

### Search Strategy

A systematic search was conducted in PubMed, Scopus, Web of Science, and the Cochrane Central Register of Controlled Trials (CENTRAL) for studies published from database inception to February 19th, 2025. Reference lists of all included articles were manually screened to identify additional relevant studies. The complete search strategy is provided in (Supplementary file 1).

### Eligibility Criteria and Study Selection

We included all randomized clinical trials (RCTs) comparing plozasiran to placebo, evaluating the change in triglycerides and APO C-III levels at the end of the study in patients with Dyslipidemic disorders. All observational studies, cross-sectional, cohort, case-control studies, non-English, in vitro, animal studies, reviews, editorials, expert opinions, conference abstracts, study protocols, and case reports were excluded. Two independent authors conducted the study screening process, and the conflicts were resolved by discussion and a third author’s opinion.

### Data Extraction

Two independent authors extracted the data in a standardized Excel sheet, including studies’ characteristics (e.g. author, date, study design), patients’ demographics, baseline characteristics as co-morbidities, Metabolic profile; baseline triglyceride level, APOC-III Level, concomitant medication use and outcomes of interest. Numerical conversions were carried out using the recently developed meta-analysis accelerator tool [20].

### Quality Assessment

The quality of randomized controlled trials was assessed using the **Cochrane Risk of Bias 2.0 (RoB 2)** tool [21]. Two reviewers independently performed the assessments, and discrepancies were resolved through consensus.

### Outcomes

The primary efficacy outcomes were percentage changes from baseline in triglyceride (TG) and apolipoprotein C-III (APOC-III) levels at 24 weeks and the end of the study. Secondary efficacy outcomes assessed percentage changes from baseline in non-high-density lipoprotein cholesterol (non-HDL), high-density lipoprotein cholesterol (HDL-C), low-density lipoprotein cholesterol (LDL-C), and apolipoprotein B (ApoB) at 24 weeks and the end of the study. Safety outcomes included any adverse events, any serious adverse events, adverse events leading to discontinuation, COVID-19, upper respiratory tract infection (URTI), headache, absolute change from baseline in platelet count at the end of the study, and absolute changes from baseline in aspartate aminotransferase (AST), alanine aminotransferase (ALT), and glycated hemoglobin (HbA1C) at 24 weeks and the end of the study.

### Statistical Analysis

Statistical analysis was conducted using RevMan software Version 5.4.1. Risk ratio (RR) was calculated for RCTs using raw counts for dichotomous data. The mean difference (MD) with 95% confidence intervals (CI) was calculated for continuous data. Statistical significance was indicated by a p-value below 0.05. A random-effects model was used when the chi-square P-value was less than 0.1, followed by a leave-one-out test to identify studies accountable for observed heterogeneity. Lastly, a subgroup analysis based on drug dosage and regimen was conducted in outcomes exhibiting persistent data heterogeneity. Subgroups were divided according to doses into 10 mg, 25 mg, 50 mg, and 100 mg, and according to regimen into monthly (M), quarterly (Q), and half-yearly (H). The results of the subgroup analyses at 24 weeks are available in the Supplementary File 2. Publication bias could not be assessed due to the limited number of included studies in this meta-analysis.

## Results

### Literature Search Results

**Fig. 1:**
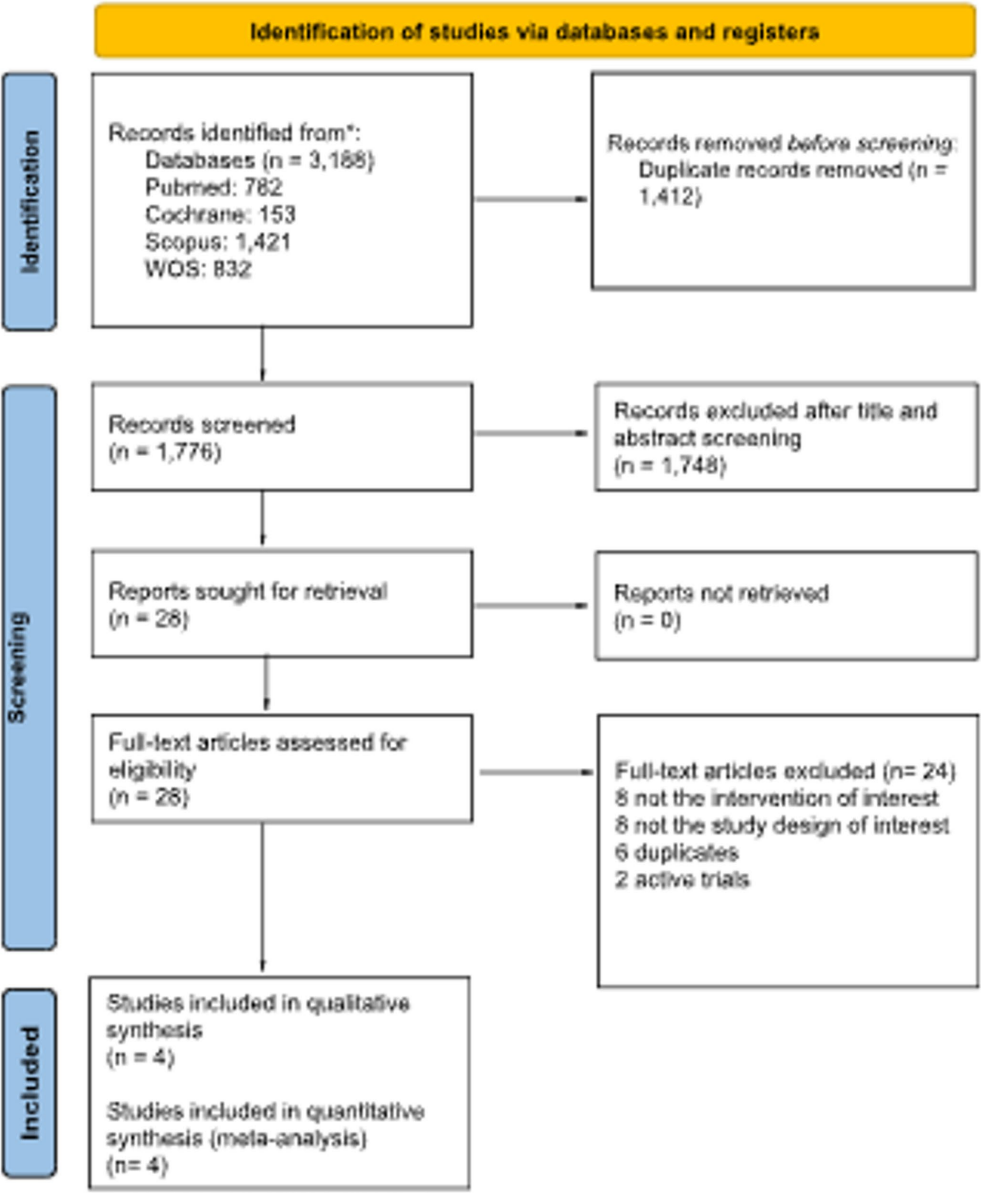
Prisma flow diagram

Our comprehensive database search initially identified 3,188 studies. After removing 1,412 duplicates, 1,776 records remained for title and abstract screening. Following this, 1,748 studies were excluded as they did not meet the inclusion criteria. Of the 28 articles selected for full-text review, 24 were subsequently excluded after evaluation. Ultimately, four RCTs met al.l eligibility criteria and were included in the final analysis [16, 17, 22, 23]. A detailed breakdown of the study selection process is provided in the PRISMA flow diagram (Fig. 1).

### Characteristics of Individual Studies

The meta-analysis included data from 4 clinical trials [16, 17, 22, 23], encompassing a total of 1,514 participants—534 assigned to various doses and treatment durations of plozasiran, and 980 receiving placebo. Approximately 63% of participants across all studies were male, with a mean age of 55.1 years. Baseline triglyceride concentrations varied widely, ranging from 234.1 mg/dL to 2,491.5 mg/dL, reflecting the inclusion of individuals with diverse lipid profiles. Similarly, baseline APOC-III levels ranged from 14.6 to 51.1 mg/dL. The trials were conducted across multiple centers in the United States, Europe, New Zealand, Australia, and Canada. Comprehensive details of each study are presented in Table 1, while participant baseline characteristics are summarized in Table 2.

**Table 1:** Comprehensive overview of the included studies

Study ID	Country, site involved	Type of the study	Sample size of each group	Inclusion criteria	Exclusion criteria	Intervention group drugs and doses	Control group drugs and doses	study duration	conclusion
Ballantyne 2024	This trial was conducted at 36 centers across the United States, Europe, New Zealand, Australia, and Canada	Phase 2b RCT	Pooled Placebo (*N* = 87) Plozasiran,10 mg Quarterly (*N* = 67) Plozasiran,25 mg Quarterly (*N* = 67) Plozasiran,50 mg Quarterly (*N* = 66) Plozasiran,50 mg Half-Yearly(*N* = 66)	1. Age: Men or nonpregnant, nonlactating women (≥ 18 years old). 2.Fasting Triglyceride Level: 150 to 499 mg/dL (1.69 to 5.63 mmol/L). 3.LDL Cholesterol: ≥70 mg/dL (≥ 1.81 mmol/L) or Non-HDL Cholesterol: ≥100 mg/dL (≥ 2.59 mmol/L). 3.Diet Stability: Maintained a stable diet for at least 2 weeks. 4.Statin Use: On a stable, maximally tolerated statin dose for at least 4 weeks (unless unable or unwilling to take statins). 5.Background Medications: Receiving stable doses of other medications.	1. Current use or use within last 365 days of any hepatocyte targeted siRNA or antisense oligonucleotide molecule; 2. Active pancreatitis within 12 weeks prior to Day 1 3. Any planned bariatric surgery or coronary intervention during the study 4. History of acute coronary syndrome event within 24 weeks of Day 1; 5. New York Heart Association (NYHA) Class II, III, or IV heart failure or last known ejection fraction of < 30%; 6. Uncontrolled hypertension (blood pressure > 160/100 mmHg at Screening), 7. History of hemorrhagic stroke within 24 weeks of Day 1; 8. History of bleeding diathesis or coagulopathy; 9. Current diagnosis of nephrotic syndrome; 10. Any of the followingLiver Function: ALT or AST > 2× ULN. Kidney Function: eGFR < 30 mL/min/1.73 m² (MDRD equation). Diabetes Control: HbA1c > 9.0% (or > 75 mmol/mol IFCC units). Proteinuria: Persistent protein > 1 + on urine dipstick (≥ 2 consecutive tests). Coagulation: Clinically significant abnormality in PT, aPTT, or INR. 11. Systemic use of corticosteroids or anabolic steroids 12. HIV, HBV, or HCV infection 13. Blood donation of 50 to 499 mL within 4 weeks of Screening 14. Clinical evidence of uncontrolled hypothyroidism or hyperthyroidism, 15. History of malignancy within the last 2 years except for adequately treated basal cell carcinoma, squamous cell skin cancer, superficial bladder tumors, or in situ cervical cancer. 16. Unwilling to limit alcohol consumption to within moderate limits for the duration of the study,	Plozasiran,10 mg Quarterly (Day 1 and week 12) Plozasiran,25 mg Quarterly (Day 1 and week 12) Plozasiran,50 mg Quarterly (Day 1 and week 12) Plozasiran,50 mg Half-Yearly (Day 1 and week 12)	Matched Placebo	From September 28, 2021, to August 14, 2023 (685 days)	In this randomized, controlled trial involving participants with mixed hyperlipidemia, plozasiran, as compared with placebo, significantly reduced triglyceride levels at 24 weeks. A clinical outcomes trial is warranted.
Gaudet 2023	This trial was conducted at centers across Australia (four sites), Canada (one site), and New Zealand (three sites)	Phase 1 RCT	Pooled Placebo (*N* = 8) Hypertriglyceridemia: Plozasiran (ARO-APOC3),10 mg (*N* = 8) Plozasiran (ARO-APOC3),25 mg (*N* = 8) Plozasiran (ARO-APOC3),50 mg (*N* = 8) Plozasiran (ARO-APOC3),100 mg (*N* = 8) Chylomicronemia: Plozasiran (ARO-APOC3),50 mg (*N* = 20)	1.Males and non-nursing females aged 18 to 70 years 2.Body mass index (BMI): 19.0 to 40.0 kg/m² 3.Stable diet for at least 4 weeks with no plans to significantly change diet or BMI during the trial 4.Screening fasting triglycerides ≥ 300 mg/dL (≥ 3.38 mmol/L) on at least one screening blood test 5.Diagnosis of familial chylomicronemia syndrome (FCS) or chylomicronemia based on: Source-verifiable records, or Fasting serum triglycerides at screening of ≥ 880 mg/dL (≥ 9.94 mmol/L) 6.No upper limit for serum triglycerides in the inclusion criteria 7.Must use two highly effective forms of contraception (for both male and female partners) during the study and for 3 months after the ARO-APOC3 dose 8.AST and ALT < 3× ULN (upper limit of normal) at screening	1. Female participants with a positive pregnancy test or who are lactating 2. Acute signs of hepatitis 3. Seropositive for HBV or HCV Medications & Substances: 4.Use of prescription medication that, in the opinion of the Principal Investigator (PI) or Sponsor, would interfere with the study (Use of antiplatelet or anticoagulant medication is acceptable) 5.Regular alcohol use within one month before screening 6. HIV infection 7. Uncontrolled hypertension (blood pressure > 170/100 mmHg) 8. Family history of: Congenital long QT syndrome Brugada syndrome Unexplained sudden cardiac death 9. Symptomatic heart failure (per New York Heart Association guidelines) 10. Severe cardiovascular disease, including: Ejection fraction < 20% Unstable angina Myocardial infarction within 6 months before study entry Transient ischemic attack (TIA) or cerebrovascular accident (CVA) within 6 months before study entry (Stable known cardiovascular disease is acceptable) 10.History of major surgery within 3 months before screening 11.History of malignancy within the last 2 years, except for: Adequately treated basal cell carcinoma Squamous cell skin cancer Superficial bladder tumors In situ cervical cancer 12. History of clinically meaningful coagulopathy or bleeding diathesis Stroke or myocardial infarction within 6 months before baseline 13. Creatinine > 1.5 mg/dL (> 132 µmol/L) at screening	Hypertriglyceridemia: Plozasiran (ARO-APOC3),10 mg (Days 1 and 29) Plozasiran (ARO-APOC3),25 mg (Days 1 and 29) Plozasiran (ARO-APOC3),50 mg (Days 1 and 29) Plozasiran (ARO-APOC3),100 mg (Days 1 and 29) Chylomicronemia: Plozasiran (ARO-APOC3),50 mg (Days 1 and 29)	Matched Placebo	113 days	In this small trial of short duration, ARO-APOC3 was associated with few AEs and reduced serum levels of APOC3 and triglycerides in healthy participants and patients with hypertriglyceridemia.
Gaudet 2024	This trial was conducted at 74 centers across the US, Europe, New Zealand, Australia, and Canada	Phase 2b RCT	Pooled Placebo (*N* = 60) Plozasiran 10 mg (*N* = 54) Plozasiran 25 mg (*N* = 55) Plozasiran 50 mg (*N* = 57)	1. Males or nonpregnant (who do not plan to become pregnant), nonlactating females ≥18 years of age 2. Based on medical history, prior evidence of fasting TG ≥ 500 mg/dL (5.65 mmol/L); 3. A mean fasting TG ≥ 500 mg/dL (5.65 mmol/L) and ≤ 4000 mg/dL (45.2 mmol/L) collected at two separate and consecutive visits at least 7 days apart and no more than 17 days apart during the Screening period; 4. If the participant has a medical history of clinical Atherosclerotic Cardiovascular Disease or elevated 10-year Atherosclerotic Cardiovascular Disease risk then the participant must be on appropriate lipid-lowering therapy as per local standard of care prior to collection of qualifying TG levels; 5. Participants of childbearing potential must agree to use highly-effective contraception, during the study and for at least 24 weeks following the last dose of IP. Males must not donate sperm during the study and for at least 24 weeks following the or last dose of IP	1. Was using or used within the last 365 days from Day 1 any hepatocyte-targeted siRNA or antisense oligonucleotide molecule 2. Active pancreatitis within 12 weeks prior to Day 1; 3. Known genetically confirmed diagnosis of Familial Chylomicronemia Syndrome 4. Any planned bariatric surgery or similar procedures to induce weight loss during the period starting at consent through end of the study; 5. History of major surgery within 12 weeks of Day 1 or planned major surgery during the study; 6. Planned coronary intervention or history of acute coronary syndrome event within 24 weeks of Day 1 7. New York Heart Association (NYHA) Class II, III, or IV heart failure 8. Uncontrolled hypertension (sitting blood pressure > 160/100 mmHg at Screening) 9. History of hemorrhagic stroke within 24 weeks of Day 1 or history of bleeding diathesis or coagulopathy 10. Medical Conditions: ASCVD, nephrotic syndrome, severe liver/kidney disease (eGFR < 30), uncontrolled diabetes (HbA1c > 9%), thyroid disorders, severe proteinuria, or recent cancer (past 2 years). 11. Infections: HIV, Hepatitis B or C. 12. Medications/Treatments: Recent use of corticosteroids, anabolic steroids, plasma apheresis, investigational drugs, or prior ARO-APOC3/ARO-ANG3 exposure (< 1 year). 13. Recent blood donation, excessive alcohol intake, or any condition affecting study compliance/safety	Plozasiran,10 mg (Day 1 and week 12) Plozasiran,25 mg (Day 1 and week 12) Plozasiran,50 mg (Day 1 and week 12)	Matched Placebo	From May 31, 2021, to August 31, 2023 (822 days) (first 336 days(48 weeks) were blinded and the rest was open label)	In this randomized clinical trial of patients with sHTG, plozasiran decreased triglyceride levels, which fell below the 500 mg/dL threshold of acute pancreatitis risk in most participants. Other triglyceride-related lipoprotein parameters improved. An increase in LDL-C level was observed but with no change in ApoB level and a decrease in non–HDL-C level. The safety profile was generally favorable at all doses.
Watts G.F. 2025	This trial was conducted in 58 centers in 21 countries.	Phase 3 RCT	Plozasiran 25 mg (*N* = 26) Plozasiran 50 mg (*N* = 24) Placebo (*N* = 25)	1. Age ≥ 18 years 2. Diagnosis of severe hypertriglyceridemia resistant to standard lipid-lowering therapy 3. Fasting triglyceride level > 1000 mg/dL on at least three occasions 4. At least one of the following: Genetic diagnosis of familial chylomicronemia syndrome (FCS) Low postheparin lipoprotein lipase activity (< 20% of normal) History of acute pancreatitis (not due to alcohol or gallstones) Recurrent hospitalizations for severe abdominal pain (without another cause) Childhood pancreatitis Family history of hypertriglyceridemia-induced pancreatitis	1. Uncontrolled diabetes 2. Use of corticosteroids or anabolic steroids 3. Chronic kidney disease	Plozasiran 25 mg (every 3 months for 12 months) Plozasiran 50 mg (every 3 months for 12 months)	Matched Placebo	From January 2022 through April 2024 (first 12 months were blinded and the rest was open label)	Patients with persistent chylomicronemia who received plozasiran had significantly lower triglyceride levels and a lower incidence of pancreatitis than those who received placebo.

**Table 2:** Baseline characteristics of enrolled patients in each included study

Study ID	Arms	Number of patients	Age (years)	Male	BMI, kg/m2	Race				Past medical history		Metabolic profile						Current medication use			
						White	Black	Asian	Other	Diabetes	Pancreatitis	Baseline Triglyceride level	Baseline APOC-III level	Baseline Non-HDL Cholesterol level	Baseline HDL Cholesterol level	Baseline LDL Cholesterol level	Baseline ApoB level	Statins	Fibrates	ω−3 Fatty acids	PCSK9 inhibitor
			mean ± SD	n (%)	(mean ± SD)	n (%)	n (%)	n (%)	n (%)	n (%)	n (%)	(mean ± SD)	(mean ± SD)	(mean ± SD)	(mean ± SD)	(mean ± SD)	(mean ± SD)	n (%)	n (%)	n (%)	n (%)
Ballantyne 2024	Plozasiran 10 mg Quarterly	67	60.2 ± 11.7	36 (54)	30.5 ± 5.7	62 (93)	N/A	N/A	N/A	39 (58)	N/A	253.2 ± 81.4 (mg/dl)	15.5 ± 5.5 (mg/liter)	153.5 ± 42.0 (mg/dl)	42.2 ± 11.1 (mg/dl)	105.1 ± 37.0 (mg/dl)	102.6 ± 23.0 (mg/dl)	61 (91)	10 (15)	2 (3)	3 (4)
	Plozasiran 25 mg Quarterly	67	61.3 ± 11.3	37 (55)	32.4 ± 6.7	60 (90)	N/A	N/A	N/A	41 (61)	N/A	234.1 ± 72.7 (mg/dl)	15.6 ± 5.5 (mg/liter)	147.7 ± 48.4 (mg/dl)	44.7 ± 13.6 (mg/dl)	101.6 ± 43.4 (mg/dl)	100.9 ± 27.2 (mg/dl)	61 (91)	6 (9)	1 (1)	3 (4)
	Plozasiran 50 mg Quarterly	66	62.6 ± 10.5	37 (56)	32.6 ± 6.5	63 (95)	N/A	N/A	N/A	45 (68)	N/A	250.3 ± 81.3 (mg/dl)	15.0 ± 5.7 (mg/liter)	151.8 ± 49.3 (mg/dl)	42.7 ± 11.7 (mg/dl)	103.0 ± 39.7 (mg/dl)	100.6 ± 27.6 (mg/dl)	60 (91)	6 (9)	1 (2)	0 (0)
	Plozasiran 50 mg Half - yearly	66	61.3 ± 11.8	43 (65)	32.0 ± 5.6	62 (94)	N/A	N/A	N/A	39 (59)	N/A	248.0 ± 80.6 (mg/dl)	15.0 ± 5.5 (mg/liter)	153.0 ± 42.7 (mg/dl)	40.8 ± 12.6 (mg/dl)	105.6 ± 31.8 (mg/dl)	104.5 ± 24.2 (mg/dl)	57 (86)	9 (14)	3 (5)	1 (2)
	Palcebo	87	58.9 ± 9.7	46 (53)	31.2 ± 5.4	79 (91)	N/A	N/A	N/A	51 (59)	N/A	237.2 ± 76.2 (mg/dl)	14.6 ± 4.7 (mg/liter)	148.3 ± 43.4 (mg/dl)	42.1 ± 11.1 (mg/dl)	101.6 ± 38.7 (mg/dl)	102.3 ± 29.6 (mg/dl)	84 (97)	15 (17)	5 (6)	1 (1)
Gaudet 2023	Plozairan 10 mg	8	53.0(40–67) (Median + Range)	7(87.5)	31.9 ± 4.5	8(100)	N/A	0 (0)	0 (0)	N/A	N/A	478.5 (318–1381) (Median + Range) (mg/dl)	28.4 ± 10.3 (mg/dl)	211.5 ± 107.4 (mg/dl)	26.8 ± 8.9 (mg/dl)	N/A	N/A	6(75.0)	1(12.5)	N/A	N/A
	Plozairan 25 mg	8	55.5(36–62) (Median + Range)	6(75.0)	31.5 ± 3.4	8(100)	N/A	0 (0)	0 (0)	N/A	N/A	560.5 (344–3546) (Median + Range) (mg/dl)	41.5 ± 21.6 (mg/dl)	298.6 ± 185.2 (mg/dl)	28.8 ± 8.1 (mg/dl)	N/A	N/A	5(62.5)	3(37.5)	N/A	N/A
	Plozairan 50 mg	8	54.5(19–64) (Median + Range)	4(50.0)	30.5 ± 7.0	4(50.0)	N/A	0 (0)	0 (0)	N/A	N/A	504.0 (294–1593) (Median + Range) (mg/dl)	24.5 ± 12.6 (mg/dl)	208.8 ± 82.8 (mg/dl)	30.0 ± 7.0 (mg/dl)	N/A	N/A	2(25.0)	4(50.0.)	N/A	N/A
	Plozairan 100 mg	8	57.5(36–70) (Median + Range)	6(75.0)	32.2 ± 3.6	4(50.0)	N/A	2(25.0)	0 (0)	N/A	N/A	511.0 (283–1448) (Median + Range) (mg/dl)	30.2 ± 14.5 (mg/dl)	203.8 ± 54.3 (mg/dl)	33.3 ± 13.9 (mg/dl)	N/A	N/A	2(25.0)	2(25.0)	N/A	N/A
	Plozairan 50 mg (chylomicronemia)	20	47.5(20,65) (Median + Range)	11(55.0)	28.2 ± 5.6	15(75.0)	N/A	5(25.0)	0 (0)	N/A	N/A	1715.0 (344–5577) (Median + Range) (mg/dl)	51.1 ± 21.2 (mg/dl)	337.6 ± 218.7 (mg/dl)	17.6 ± 7.0 (mg/dl)	N/A	N/A	11(55.0)	13(65.0)	N/A	N/A
	Palcebo	8	46.5(30–68) (Median + Range)	6(75.0)	30.7 ± 5.0	5(62.5)	N/A	2(25.0)	0 (0)	N/A	N/A	357.0 (262–1746) (Median + Range) (mg/dl)	22.8 ± 7.4 (mg/dl)	167.5 ± 46.3 (mg/dl)	27.5 ± 7.8 (mg/dl)	N/A	N/A	3(37.5)	3(37.5)	N/A	N/A
Gaudet 2024	Plozairan 10 mg	54	53(10)	46(85)	33 ± 5	47(87)	2(4)	1(2)	3(6)	31(57)	6(11)	890 ± 577 (mg/dl)	33 ± 15 (mg/dl)	209 ± 74 (mg/dl)	28 ± 9 (mg/dl)	75 ± 44 (mg/dl)	103 ± 44 (mg/dl)	35(65)	26(48)	8(15)	1(2)
	Plozairan 25 mg	55	56(11)	43(78)	32 ± 5	48(87)	3(6)	2(4)	0 (0)	37(67)	8(15)	942 ± 756 (mg/dl)	34 ± 17 (mg/dl)	206 ± 91 (mg/dl)	30 ± 11 (mg/dl)	74 ± 40 (mg/dl)	103 ± 32 (mg/dl)	39(71)	24(44)	5(9)	2(4)
	Plozairan 50 mg	57	54(11)	41(72)	32 ± 5	53(93)	1(2)	3(5)	0 (0)	37(65)	11(19)	908 ± 653 (mg/dl)	32 ± 16 (mg/dl)	196 ± 88 (mg/dl)	31 ± 13 (mg/dl)	72 ± 42 (mg/dl)	110 ± 55 (mg/dl)	39(70)	28(50)	8(14)	1(2)
	Placebo	60	56(11)	46(77)	31 ± 4	55(92)	1(2)	3(5)	0 (0)	39(65)	13(22)	851 ± 507 (mg/dl)	31 ± 16 (mg/dl)	185 ± 79 (mg/dl)	30 ± 12 (mg/dl)	69 ± 39 (mg/dl)	95 ± 29 (mg/dl)	41(67)	31(51)	3(5)	3(5)
Watts G.F. 2025	Plozairan 25 mg	26	47.9 ± 14.4	12 (46)	26.1 ± 3.9	19 (73)	N/A	N/A	N/A	10 (38)	23 (89)	2349.5 ± 1374.5 (mg/dl)	38.5 ± 17.1 (mg/dl)	N/A	N/A	N/A	N/A	11 (42)	19 (73)	9 (35)	N/A
	Plozairan 50 mg	24	42.6 ± 10.9	11 (46)	25.4 ± 4.8	17 (71)	N/A	N/A	N/A	7 (29)	22 (92)	2491.5 ± 1523.2 (mg/dl)	32.5 ± 19.8 (mg/dl)	N/A	N/A	N/A	N/A	12 (50)	15 (63)	7 (29)	N/A
	Placebo	25	47.4 ± 13.9	14 (56)	25.0 ± 4.1	19 (76)	N/A	N/A	N/A	11 (44)	22 (88)	2271.9 ± 1141.4 (mg/dl)	39.9 ± 17.6 (mg/dl)	N/A	N/A	N/A	N/A	11 (44)	16 (64)	6 (24)	N/A

### Quality Assessment

The Cochrane RoB 2.0 tool was used to assess the risk of bias in the included RCTs. All studies were found to have a low overall risk of bias, with adequate randomization, allocation concealment, and outcome measurement and analysis (Supplementary file Fig. 1).

### Efficacy Outcomes

#### Percent Change from Baseline in TG Level

Our analysis demonstrated a statistically significant reduction in TG levels in the plozasiran group compared with placebo at both 24 weeks (MD = −53.22, 95% CI [−57.17, −49.26], *P* < 0.00001) and at the end of the study (MD = −53.00, 95% CI [−60.51, −45.50], *P* < 0.00001).

The 24-week analysis showed low heterogeneity (*P* = 0.30, I² = 16%). In contrast, moderate heterogeneity was observed at the end of the study (*P* = 0.05, I² = 43%), which was resolved by excluding the study by Ballantyne et al. (2024) [16] (10 mg Q) (*P* = 0.16, I² = 29%) (Supplementary file Fig. 2A, 2B).

Subgroup analysis at the end of the study, stratified by plozasiran regimen and dose, revealed statistically significant reductions in TG levels compared with placebo across all evaluated groups: **10 mg M** (MD = −57.65, 95% CI [−92.42, −22.88], *P* = 0.001), **10 mg Q** (MD = −32.28, 95% CI [−43.51, −21.05], *P* < 0.00001), **25 mg M** (MD = −69.55, 95% CI [−102.45, −36.65], *P* < 0.0001), **25 mg Q** (MD = −48.85, 95% CI [−59.33, −38.37], *P* < 0.00001), **50 mg M** (MD = −78.85, 95% CI [−111.62, −46.08], *P* < 0.00001), **50 mg Q** (MD = −50.63, 95% CI [−61.32, −39.94], *P* < 0.00001), **50 mg H** (MD = −56.70, 95% CI [−69.61, −43.79], *P* < 0.00001), **100 mg M** (MD = −79.88, 95% CI [−112.78, −46.97], *P* < 0.00001). The 10 mg M, 25 mg M, 50 mg M, 50 mg H, and 100 mg M groups were based on single-study data.

**Fig. 2:**
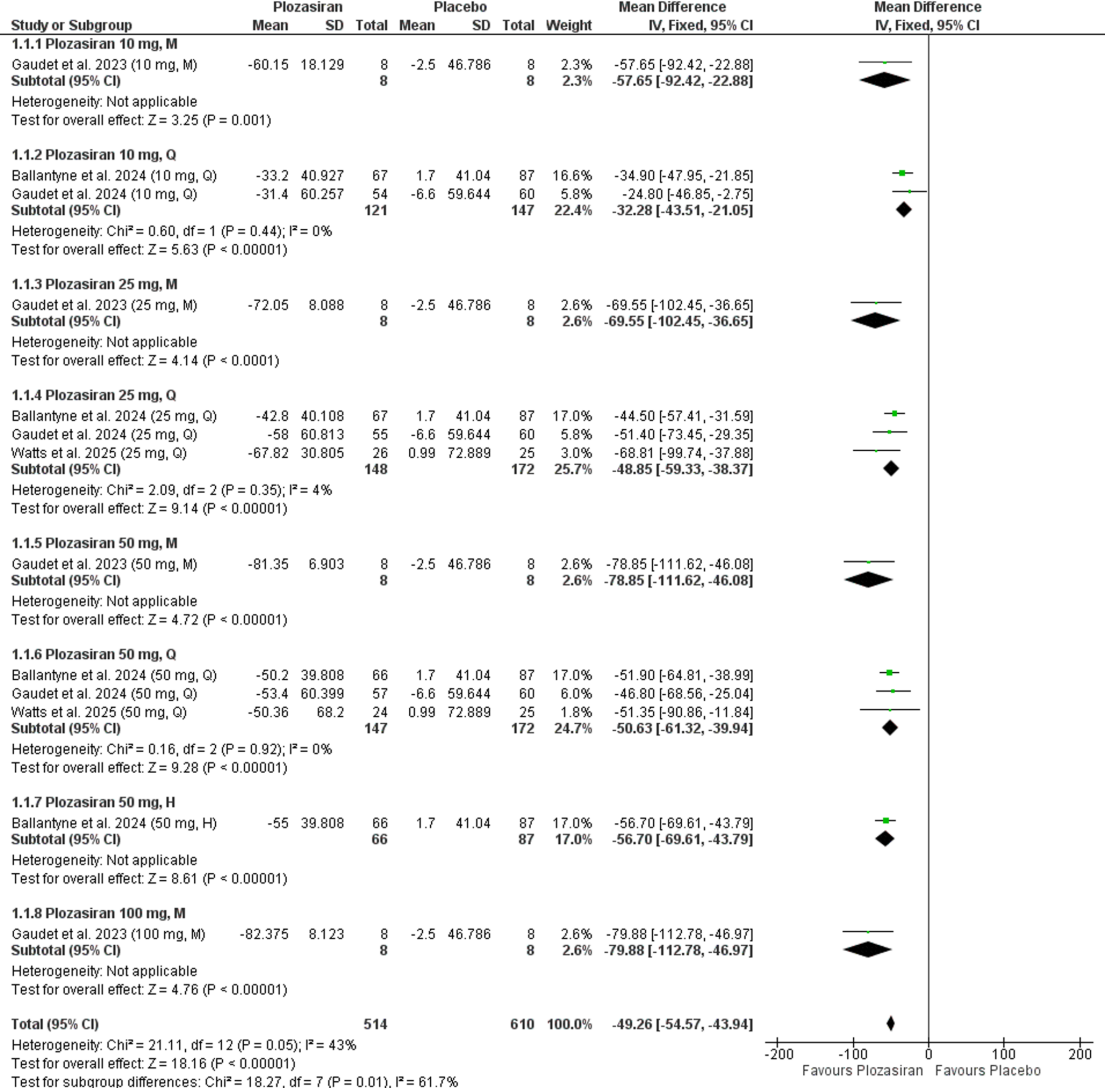
Forrest plot demonstrating percent change from baseline in TG levels at the end of the study (subgroup analysis)

Evidence of homogeneity was observed in the 10 mg Q (*P* = 0.44, I² = 0%), 25 mg Q (*P* = 0.35, I² = 4%), and 50 mg Q (*P* = 0.92, I² = 0%) subgroups. Heterogeneity assessment was not applicable for the 10 mg M, 25 mg M, 50 mg M, 50 mg H, and 100 mg M groups, as each was reported in a single study (Fig. 2).

#### Percent Change from Baseline in APOC-III Level

Our analysis demonstrated a statistically significant reduction in APOC-III levels in the plozasiran group compared to placebo at both 24 weeks (MD = −72.21, 95% CI [−79.58, −64.83], *P* < 0.00001) and at the end of the study (MD = −65.42, 95% CI [−76.04, −54.81], *P* < 0.00001).

Both analyses at 24 weeks (*P* < 0.0001, I² = 76%) and at the end of the study (*P* < 0.0001, I² = 82%) exhibited significant heterogeneity, which was not resolved by the leave-one-out test (Supplementary file Fig. 3A, 3B).

Subgroup analysis at the end of the study, stratified by plozasiran regimen and dose, revealed statistically significant reductions in APOC-III levels compared to placebo in the following groups: **10 mg M** (MD = −60.40, 95% CI [−86.50, −34.30], *P* < 0.00001), **10 mg Q** (MD = −37.58, 95% CI [−47.75, −27.41], *P* < 0.00001), **25 mg M** (MD = −80.10, 95% CI [−105.16, −55.04], *P* < 0.0001), **25 mg Q** (MD = −65.56, 95% CI [−73.66, −57.45], *P* < 0.00001), **50 mg M** (MD = −88.50, 95% CI [−113.14, −63.86], *P* < 0.00001), **50 mg Q** (MD = −68.64, 95% CI [−76.60, −60.67], *P* < 0.00001), **50 mg H** (MD = −70.30, 95% CI [−82.74, −57.86], *P* < 0.00001), **100 mg M** (MD = −92.80, 95% CI [−117.08, −68.52], *P* < 0.00001). The 10 mg M, 25 mg M, 50 mg M, 50 mg H, and 100 mg M groups were based on single-study data.

**Fig. 3:**
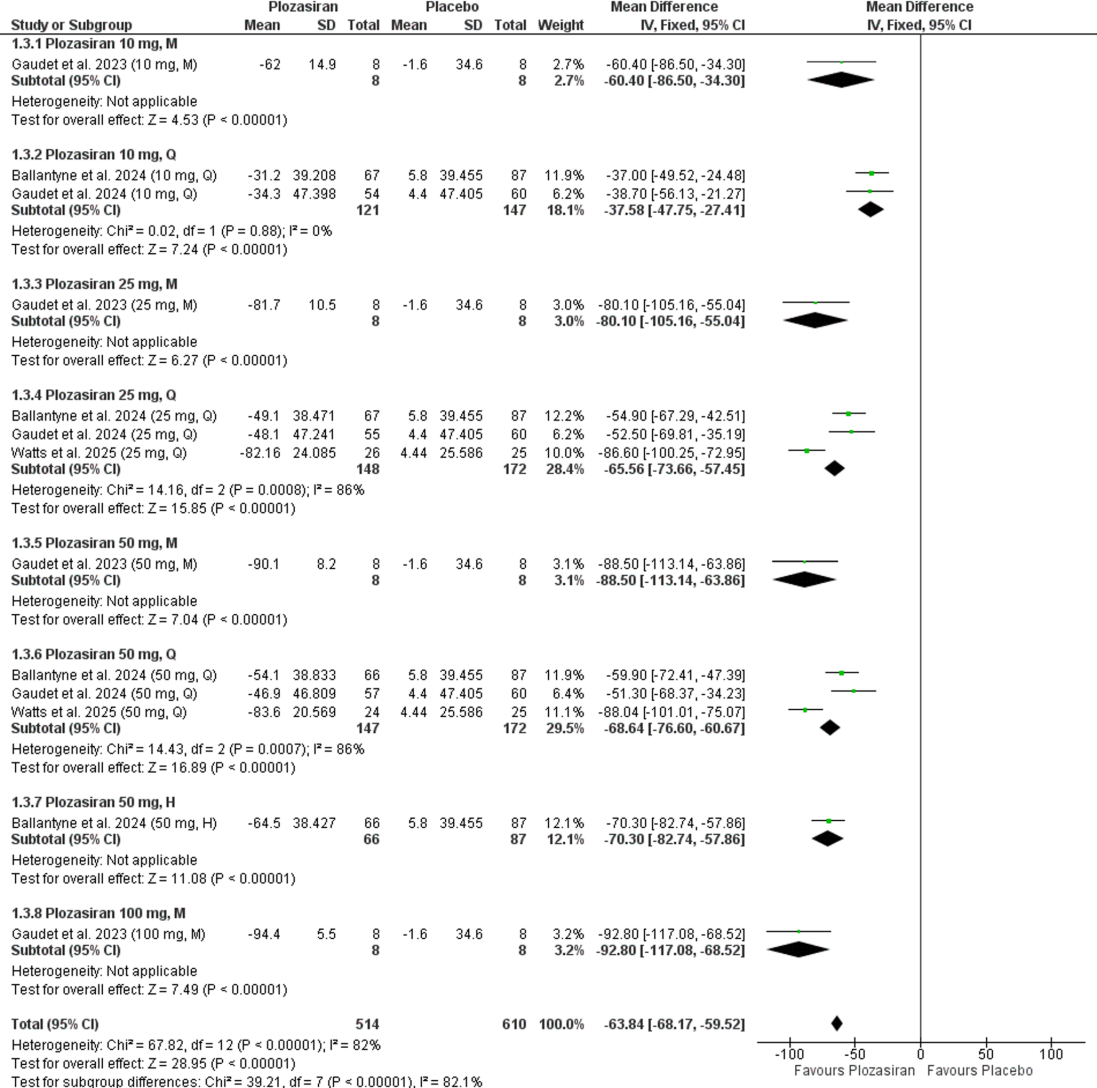
Forrest plot illustrating percent change from baseline in APOC-III levels at the end of the study (subgroup analysis)

Further analysis revealed statistical homogeneity in the **10 mg Q** group (*P* = 0.88, I² = 0%). However, heterogeneity was observed in the **25 mg Q** (*P* = 0.0008, I² = 86%) and **50 mg Q** (*P* = 0.0007, I² = 86%) subgroups. Heterogeneity assessments were not applicable for the **10 mg M**, **25 mg M**, **50 mg M**, **50 mg H**, and **100 mg M** groups, as these outcomes were reported in a single study (Fig. 3).

#### Percent Change from Baseline in HDL-C Level

Our analysis demonstrated a statistically significant increase in HDL-C levels in the plozasiran group compared to placebo at both 24 weeks (MD = 43.99, 95% CI [37.51, 50.47], *P* < 0.00001) and at the end of the study (MD = 36.73, 95% CI [27.79, 45.67], *P* < 0.00001).

At 24 weeks, the analysis showed significant heterogeneity (*P* = 0.02, I² = 60%), which was resolved by excluding the study by Ballantyne et al. (2024) [16] (50 mg, H) (*P* = 0.15, I² = 38%). Similarly, at the end of the study, heterogeneity remained significant (*P* < 0.00001, I² = 77%) but was not resolved by the leave-one-out test (Supplementary file Fig. 4A, 4B).

Subgroup analysis at the end of the study, stratified by regimen and plozasiran dose, revealed statistically significant increases in HDL-C levels compared to placebo in the following groups: **10 mg M** (MD = 41.90, 95% CI [19.26, 64.54], *P* = 0.0003), **10 mg Q** (MD = 19.80, 95% CI [11.11, 28.49], *P* < 0.00001), **25 mg M** (MD = 50.00, 95% CI [37.79, 62.21], *P* < 0.0001), **25 mg Q** (MD = 26.09, 95% CI [17.46, 34.73], *P* < 0.00001), **50 mg M** (MD = 84.80, 95% CI [51.73, 117.87], *P* < 0.00001), **50 mg Q** (MD = 30.42, 95% CI [21.82, 39.01], *P* < 0.00001), **50 mg H** (MD = 40.00, 95% CI [29.53, 50.47], *P* < 0.00001), **100 mg M** (MD = 81.10, 95% CI [53.55, 108.65], *P* < 0.00001). The 10 mg M, 25 mg M, 50 mg M, 50 mg H, and 100 mg M groups were based on single-study data.

**Fig. 4:**
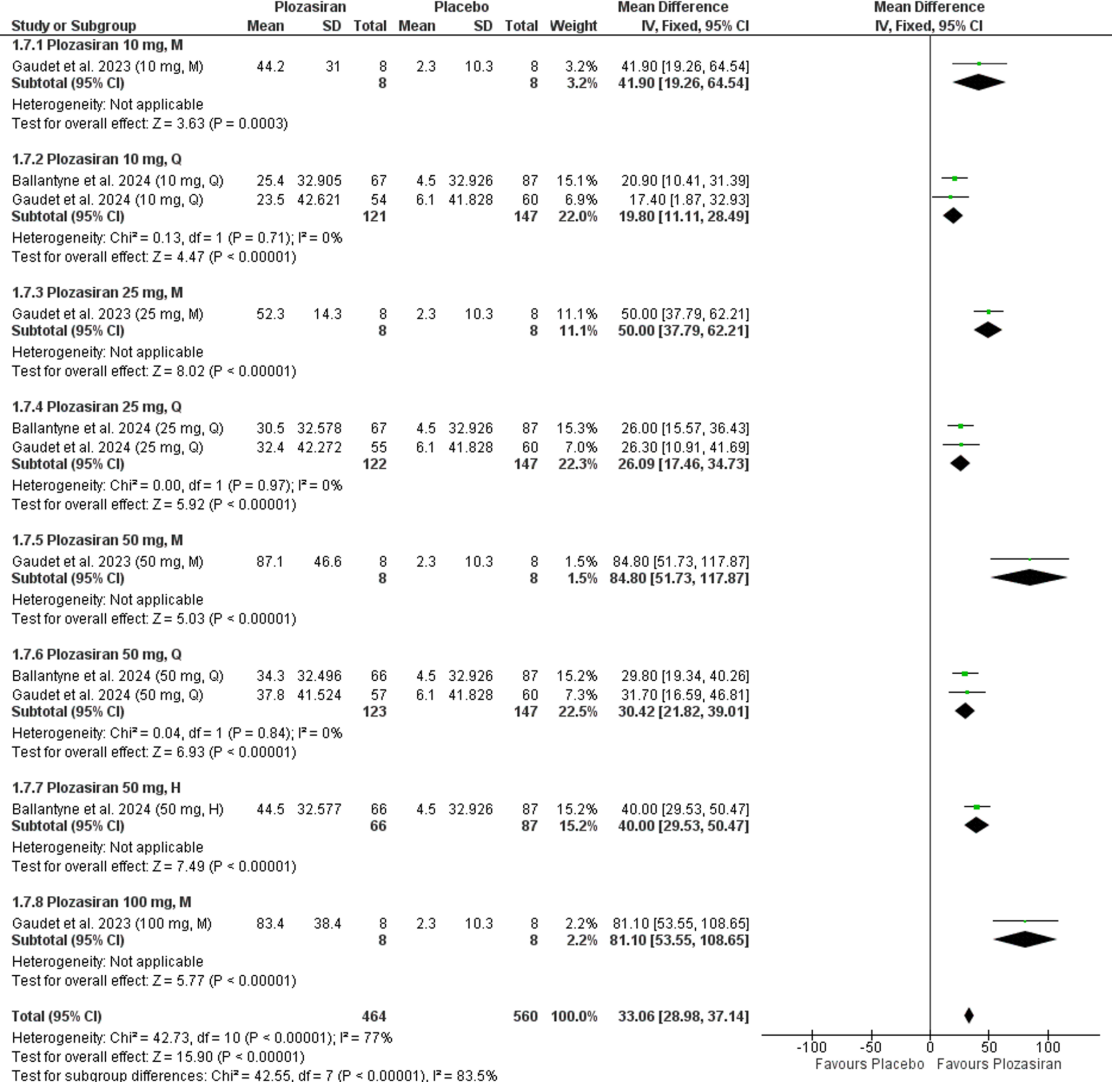
Forrest plot illustrating percent change from baseline in HDL-C levels at the end of the study (subgroup analysis)

Further analysis revealed homogeneity in the following groups: **10 mg Q** (*P* = 0.71, I² = 0%), **25 mg Q** (*P* = 0.97, I² = 4%), **50 mg Q** (*P* = 0.84, I² = 0%). Heterogeneity assessment was not applicable for the **10 mg M**, **25 mg M**, **50 mg M**, **50 mg H**, and **100 mg M** groups, as these outcomes were reported in a single study (Fig. 4).

#### Percent Change from Baseline in LDL-C Level

Our analysis demonstrated no statistically significant difference in LDL-C levels in the plozasiran group compared to placebo at both 24 weeks (MD = 7.65, 95% CI [−4.49, 19.80], *P* = 0.22) and at the end of the study (MD = −2.06, 95% CI [−7.01, 2.88], *P* = 0.41).

At 24 weeks, the analysis exhibited significant heterogeneity (*P* < 0.00001, I² = 81%), which was not resolved by the leave-one-out test. In contrast, homogeneity was observed at the end of the study (*P* = 0.18, I² = 28%) (Supplementary file Fig. 5A, 5B).

Subgroup analysis at the end of the study, stratified by regimen and plozasiran dose, showed no statistically significant difference in LDL-C levels compared to placebo in the following groups: **10 mg M** (MD = 11.40, 95% CI [−33.56, 56.24], *P* = 0.62), **10 mg Q** (MD = −1.14, 95% CI [−11.05, 8.77], *P* = 0.82), **25 mg M** (MD = 146.70, 95% CI [−48.38, 341.78], *P* = 0.14), **25 mg Q** (MD = 2.53, 95% CI [−7.24, 12.31], *P* = 0.61), **50 mg M** (MD = 59.50, 95% CI [−42.33, 116.33], *P* = 0.25), **50 mg Q** (MD = −7.02, 95% CI [−16.87, 2.83], *P* = 0.16), **50 mg H** (MD = −5.20, 95% CI [−15.64, 5.24], *P* = 0.33), **100 mg M** (MD = 86.50, 95% CI [−37.33, 210.33], *P* = 0.17). The 10 mg M, 25 mg M, 50 mg M, 50 mg H, and 100 mg M groups were based on single-study data.

Further analysis revealed homogeneity in the following groups: **10 mg Q** (*P* = 0.37, I² = 0%), **25 mg Q** (*P* = 0.47, I² = 0%). Heterogeneity was observed in the **50 mg Q** group (*P* = 0.04, I² = 77%), and heterogeneity assessments were not applicable for the **10 mg M**, **25 mg M**, **50 mg M**, **50 mg H**, and **100 mg M** groups, as these outcomes were reported in a single study (Supplementary file Fig. 5C).

#### Percent Change from Baseline in Non-HDL Cholesterol Level

Our analysis demonstrated a statistically significant reduction in Non-HDL cholesterol levels in the **plozasiran** group compared to placebo, both at 24 weeks (MD = −21.35, 95% CI [−25.14, −17.55], *P* < 0.00001) and at the end of the study (MD = −21.56, 95% CI [−26.69, −16.42], *P* < 0.00001).

At 24 weeks, the analysis exhibited significant heterogeneity (*P* = 0.02, I² = 62%), which was resolved after excluding Ballantyne et al., 2024 [16] (50 mg, H) (*P* = 0.35, I² = 10%). In contrast, heterogeneity was still observed at the end of the study (*P* = 0.04, I² = 48%), but was not resolved by the leave-one-out test (Supplementary file Fig. 6A, 6B).

In the subgroup analysis at the end of the study, stratified by regimen and doses of plozasiran, compared to placebo, there were statistically significant reductions in Non-HDL cholesterol levels in the following groups:**10 mg M** (MD = −32.00, 95% CI [−48.37, −15.63], *P* = 0.0001), **10 mg Q** (MD = −12.78, 95% CI [−20.10, −5.47], *P* = 0.0006), **25 mg M** (MD = −39.40, 95% CI [−58.92, −19.88], *P* < 0.0001), **25 mg Q** (MD = −16.78, 95% CI [−24.00, −9.56], *P* < 0.00001), **50 mg M** (MD = −39.40, 95% CI [−58.12, −20.68], *P* < 0.00001), **50 mg Q** (MD = −20.50, 95% CI [−27.74, −13.25], *P* < 0.00001), **50 mg H** (MD = −21.70, 95% CI [−30.46, −12.94], *P* < 0.00001), **100 mg M** (MD = −32.40, 95% CI [−51.30, −13.50], *P* = 0.0008). The 10 mg M, 25 mg M, 50 mg M, 50 mg H, and 100 mg M groups were based on single-study data.

**Fig. 5:**
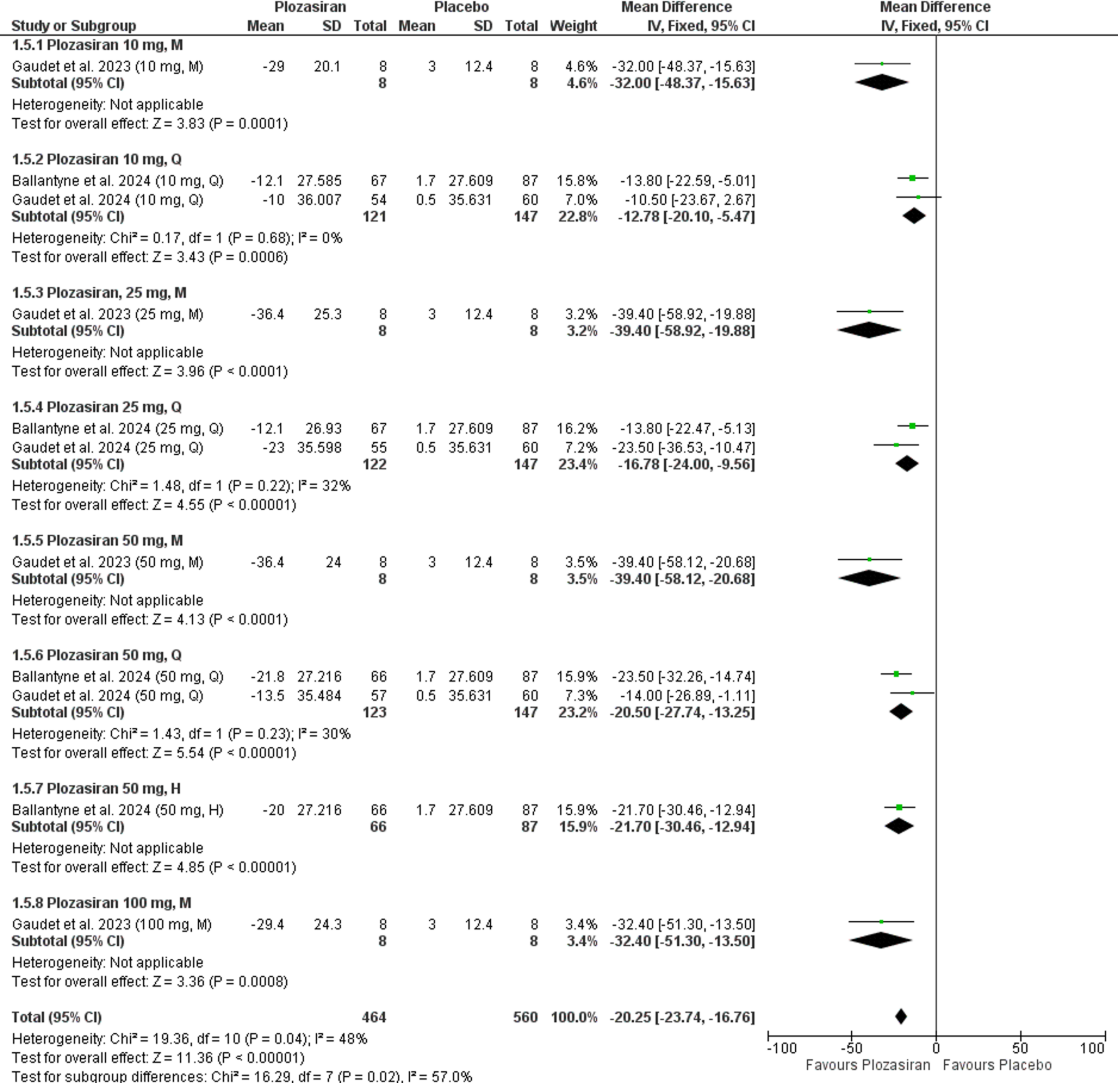
Forest plot showing percent change from baseline in Non-HDL cholesterol levels at the end of the study (subgroup analysis)

Further analyses revealed homogeneity in the following groups: **10 mg Q** (*P* = 0.68, I² = 0%), **25 mg Q** (*P* = 0.22, I² = 32%), **50 mg Q** (*P* = 0.23, I² = 30%). However, homogeneity could not be assessed for the **10 mg M**, **25 mg M**, **50 mg M**, **50 mg H**, and **100 mg M** groups, as the outcome was reported in only one study (Fig. 5).

#### Percent Change from Baseline in ApoB Level

Our analysis demonstrated a statistically significant reduction in Apo B levels in the plozasiran group compared to placebo at both 24 weeks (MD = −11.61, 95% CI [−15.15, −8.07], *P* < 0.00001) and at the end of the study (MD = −9.89, 95% CI [−13.19, −6.59], *P* < 0.00001). Both analyses at 24 weeks (*P* = 0.30, I² = 17%) and at the end of the study (*P* = 0.50, I² = 0%) showed homogeneity (Supplementary file Fig. 7A, 7B).

In the subgroup analysis at the end of the study, stratified by regimen and doses of **plozasiran**, compared to placebo, statistically significant reductions in Apo B levels were observed in the following groups:**25 mg Q** (MD = −8.45, 95% CI [−15.05, −1.85], *P* = 0.01), **50 mg Q** (MD = −11.37, 95% CI [−17.98, −4.75], *P* = 0.0008), **50 mg H** (MD = −15.50, 95% CI [−23.64, −7.36], *P* = 0.0002). The 10 mg M, 25 mg M, 50 mg M, 50 mg H, and 100 mg M groups were based on single-study data.

Conversely, there were no significant differences observed in the following groups:**10 mg M** (MD = −13.90, 95% CI [−29.64, −1.84], *P* = 0.08), **10 mg Q** (MD = −5.13, 95% CI [−11.82, 1.57], *P* = 0.13), **25 mg M** (MD = −4.90, 95% CI [−31.31, 21.51], *P* = 0.72), **50 mg M** (MD = −13.20, 95% CI [−34.96, 8.56], *P* = 0.23), **100 mg M** (MD = −13.90, 95% CI [−42.57, 14.77], *P* = 0.34).

The analyses demonstrated homogeneity in the following groups: **10 mg Q** (*P* = 0.23, I² = 30%), **25 mg Q** (*P* = 0.60, I² = 0%), **50 mg Q** (*P* = 0.09, I² = 66%). However, homogeneity could not be assessed in the **10 mg M**, **25 mg M**, **50 mg M**, **50 mg H**, and **100 mg M** groups, as the outcome was reported in only one study (Supplementary file Fig. 7C).

### Safety Outcomes

#### Any Adverse Events

Our analysis demonstrated a statistically significant increase in the occurrence of any adverse events in the plozasiran group compared to placebo at the end of the study (RR = 1.11, 95% CI [1.03, 1.19], *P* = 0.005), with homogeneity observed (*P* = 0.96, I² = 0%) (Supplementary file Fig. 8).

In the subgroup analysis at the end of the study, stratified by regimen and doses of plozasiran, compared to placebo, we observed a statistically significant increase in any adverse events in the **50 mg Q** group (RR = 1.16, 95% CI [1.02, 1.31], *P* = 0.03). However, no significant differences were found in the following groups:**10 mg M** (RR = 1.00, 95% CI [0.57, 1.76], *P* = 1.00), **10 mg Q** (RR = 1.11, 95% CI [0.95, 1.30], *P* = 0.19), **25 mg M** (RR = 1.00, 95% CI [0.57, 1.76], *P* = 1.00), **25 mg Q** (RR = 1.03, 95% CI [0.90, 1.19], *P* = 0.64), **50 mg M** (RR = 1.31, 95% CI [0.85, 2.02], *P* = 0.23), **50 mg H** (RR = 1.17, 95% CI [0.95, 1.45], *P* = 0.14), **100 mg M** (RR = 1.00, 95% CI [0.57, 1.76], *P* = 1.00). The 10 mg M, 25 mg M, 50 mg M, 50 mg H, and 100 mg M groups were based on single-study data.

**Fig. 6:**
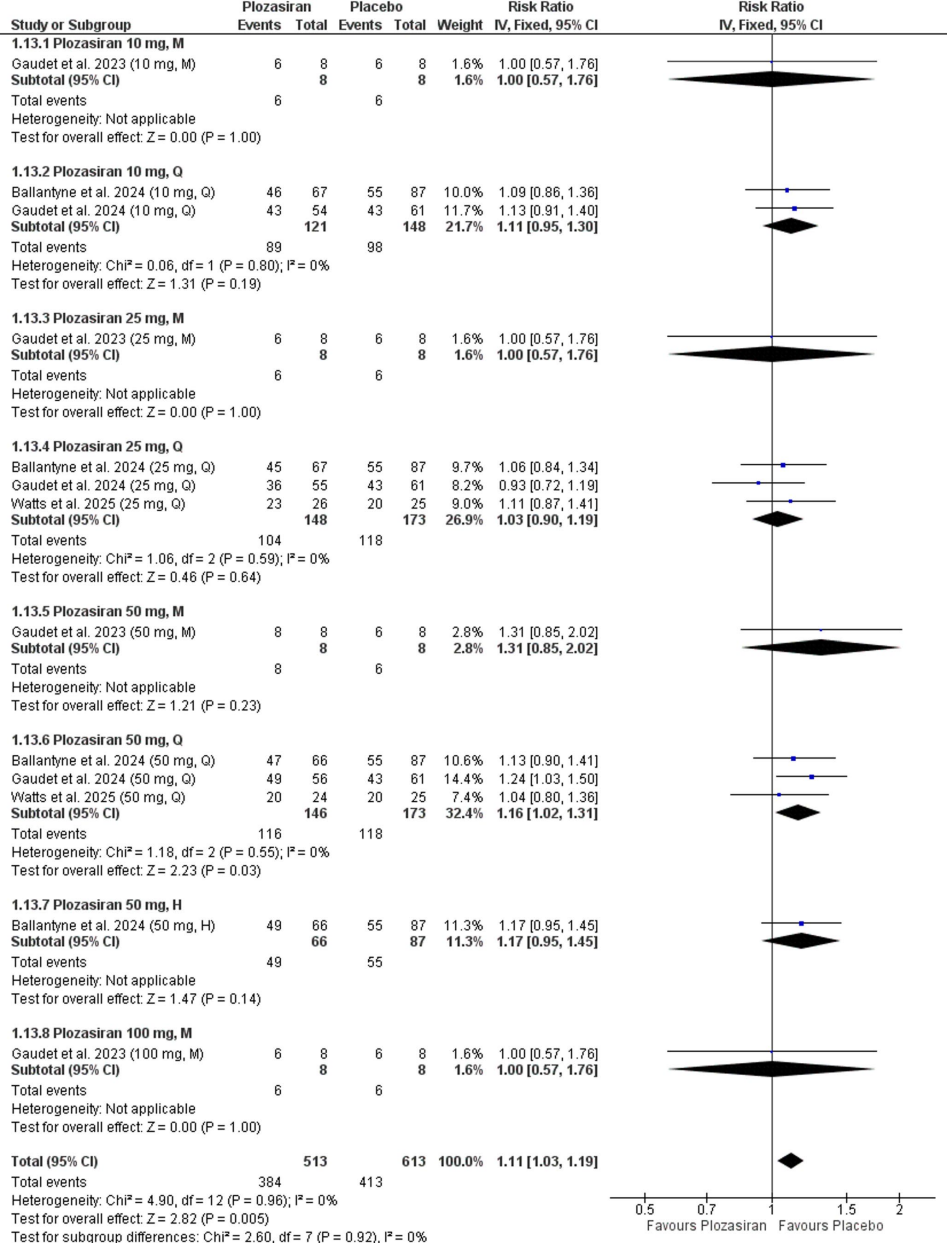
Forest plot showing any adverse events at the end of the study (subgroup analysis)

The analyses demonstrated homogeneity in the following groups: **10 mg Q** (*P* = 0.80, I² = 0%), **25 mg Q** (*P* = 0.59, I² = 0%), **50 mg Q** (*P* = 0.55, I² = 0%). However, homogeneity could not be assessed in the **10 mg M**, **25 mg M**, **50 mg M**, **50 mg H**, and **100 mg M** groups, as the outcome was reported in only one study (Fig. 6).

#### Serious Adverse Events

Our analysis showed no statistically significant difference in the occurrence of any adverse events between the polzasiran group and placebo at the end of the study (RR = 0.77, 95% CI [0.53, 1.13], *P* = 0.19), with homogeneity observed (*P* = 0.36, I² = 9%) (Supplementary file Fig. 9).

In the subgroup analysis at the end of the study, stratified by regimen and doses of plozasiran, compared to placebo, there were no statistically significant differences in serious adverse events for the following groups: **10 mg Q** (RR = 0.47, 95% CI [0.19, 1.17], *P* = 0.11), **25 mg M** (RR = 3.00, 95% CI [0.14, 64.26], *P* = 0.48), **25 mg Q** (RR = 0.66, 95% CI [0.33, 1.31], *P* = 0.24), **50 mg Q** (RR = 0.86, 95% CI [0.46, 1.60], *P* = 0.63), **50 mg H** (RR = 1.32, 95% CI [0.40, 4.37], *P* = 0.65), **100 mg M** (RR = 3.00, 95% CI [0.14, 64.26], *P* = 0.48). The 10 mg M, 25 mg M, 50 mg M, 50 mg H, and 100 mg M groups were based on single-study data.

**Fig. 7:**
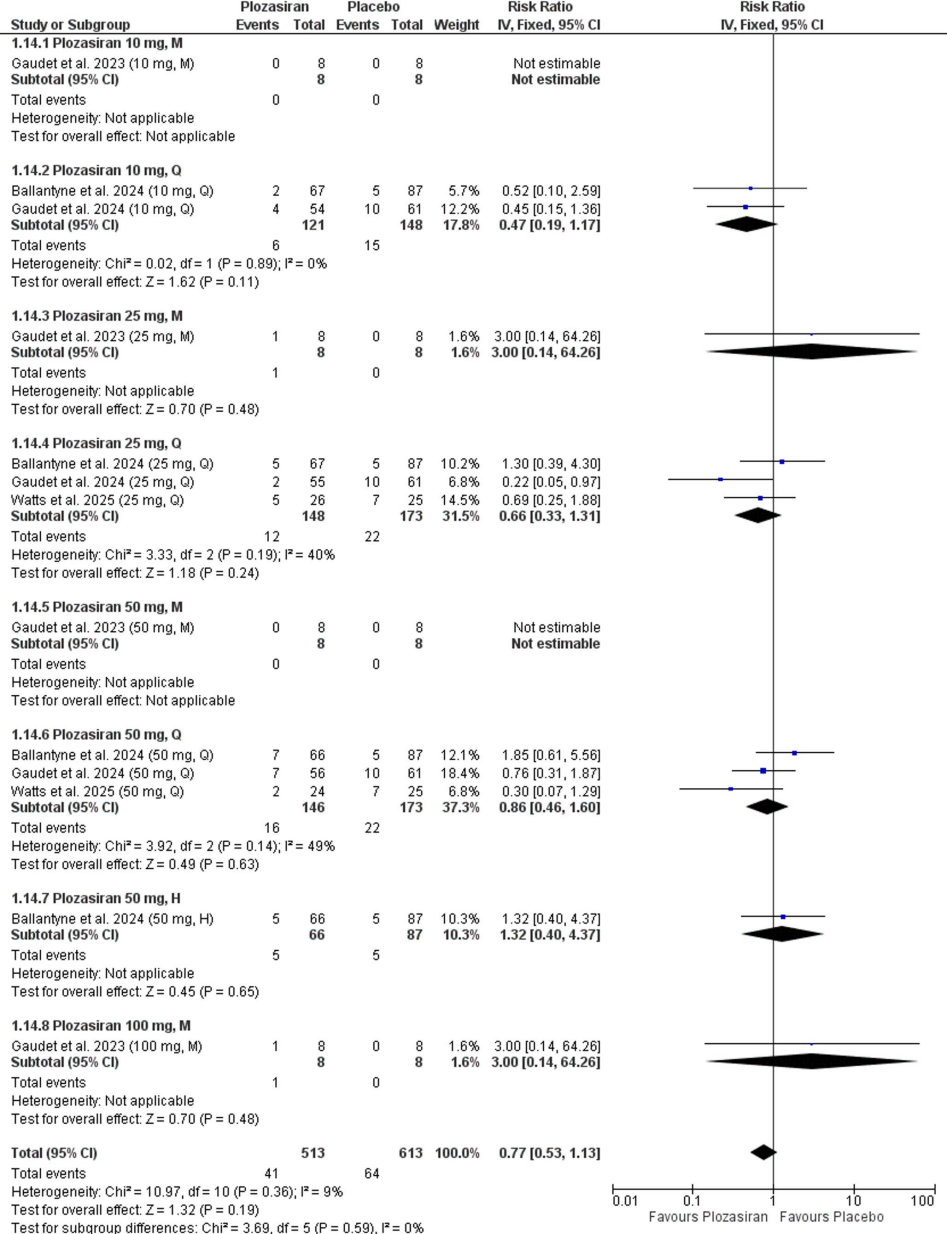
Forest plot demonstrating serious adverse events at the end of the study (subgroup analysis)

The analyses demonstrated homogeneity in the following groups: **10 mg Q** (*P* = 0.89, I² = 0%), **25 mg Q** (*P* = 0.19, I² = 40%), **50 mg Q** (*P* = 0.14, I² = 49%). However, homogeneity could not be assessed in the **10 mg M**, **25 mg M**, **50 mg M**, **50 mg H**, and **100 mg M** groups, as the outcome was reported in only one study (Fig. 7).

#### Adverse Events Leading to Discontinuation of the Drug

Our analysis revealed no statistically significant difference in adverse events leading to discontinuation of the drug between the polzasiran group and placebo at the end of the study (RR = 0.55, 95% CI [0.26, 1.15], *P* = 0.11), with homogeneity observed (*P* = 0.82, I² = 0%) (Supplementary file Fig. 10).

#### Covid-19

Our analysis found no statistically significant difference in the incidence of **COVID-19** infection between the polzasiran group and placebo at the end of the study (RR = 0.99, 95% CI [0.72, 1.37], *P* = 0.97), with homogeneity observed (*P* = 0.44, I² = 0%) (Supplementary file Fig. 11).

#### Headache

Our analysis revealed a statistically significant increase in the incidence of headache in the polzasiran group compared to placebo at the end of the study (RR = 1.85, 95% CI [1.14, 3.02], *P* = 0.01), with homogeneity observed (*P* = 0.90, I² = 0%) (Supplementary file Fig. 12).

#### Upper Respiratory Tract Infections

Our analysis found no statistically significant difference in the incidence of URTI between the polzasiran and placebo groups at the end of the study (RR = 1.17, 95% CI [0.70, 1.95], *P* = 0.56), with homogeneity observed (*P* = 0.51, I² = 0%) (Supplementary file Fig. 13).

#### Absolute Change from Baseline in HbA1C Level

Our analysis revealed a statistically significant increase in HbA1c levels in the polzasiran group compared to placebo at both 24 weeks (MD = 0.16, 95% CI [0.03, 0.30], *P* = 0.02) and at the end of the study (MD = 0.19, 95% CI [0.06, 0.31], *P* = 0.005).

At 24 weeks, the analysis was heterogeneous (*P* = 0.09, I² = 46%), but heterogeneity was resolved by excluding Gaudet et al., 2024 [23] **(50 mg**,** Q)** (*P* = 0.65, I² = 0%). In contrast, homogeneity was observed at the end of the study (*P* = 1.00, I² = 0%) (Supplementary file Fig. 14A, 14B).

#### Absolute Change from Baseline in AST Level

Our analysis revealed no statistically significant difference in AST levels between the polzasiran group and placebo at both 24 weeks (MD = 1.17, 95% CI [−0.12, 2.45], *P* = 0.07) and at the end of the study (MD = 1.15, 95% CI [−0.08, 2.38], *P* = 0.07).

At 24 weeks, the analysis was heterogeneous (*P* = 0.04, I² = 54%), but this heterogeneity was resolved by excluding Gaudet et al., 2024 [23] **(25 mg**,** Q)** (*P* = 0.14, I² = 40%). Similarly, heterogeneity was observed at the end of the study (*P* = 0.005, I² = 63%), which was resolved by excluding Ballantyne et al., 2024 [16] **(50 mg**,** Q)** (*P* = 0.35, I² = 10%) (Supplementary file Fig. 15 A, 15B).

#### Absolute Change from Baseline in ALT Level

Our analysis demonstrated a statistically significant increase in ALT levels in the polzasiran group compared to placebo, both at 24 weeks (MD = 4.81, 95% CI [3.41, 6.20], *P* < 0.00001) and at the end of the study (MD = 3.98, 95% CI [0.88, 7.08], *P* = 0.01).

At 24 weeks, the analysis was homogeneous (*P* = 0.19, I² = 31%), whereas at the end of the study, heterogeneity was observed (*P* < 0.00001, I² = 79%). However, this heterogeneity was not resolved by the leave-one-out test (Supplementary file Fig. 16 A, 16B).

#### Absolute Change from Baseline in Platelet Count

Our analysis revealed no statistically significant difference in platelet count between the polzasiran group and placebo at the end of the study (MD = 2.34, 95% CI [−3.32, 8.00], *P* = 0.42). The analysis was homogeneous (*P* = 0.17, I² = 36%) (Supplementary file Fig. 17).

## Discussion

### Summary of Findings

Our meta-analysis demonstrated that plozasiran significantly improved TGs, APOC-III, non-HDL-C, HDL-C, and ApoB levels at both 24 weeks and at the end of the study period. In contrast, its effect on LDL-C was minimal and did not reach statistical significance.

Subgroup analyses based on dose and dosing frequency revealed consistent findings. Quarterly administration of plozasiran at 10 mg, 25 mg, and 50 mg resulted in significant improvements in TGs, APOC-III, non–HDL-C, and HDL-C at both 24 weeks and at the end of the study. For ApoB, all three doses produced significant reductions at 24 weeks; however, only the 25 mg and 50 mg quarterly regimens sustained these reductions through the end of the study. Among all regimens, plozasiran 50 mg administered quarterly demonstrated the greatest efficacy across all lipid parameters at both time points. Conversely, LDL-C levels remained largely unchanged, even when stratified by dose and dosing schedule.

In terms of safety, the overall analysis indicated that patients receiving plozasiran experienced a higher incidence of adverse events, headaches, and elevated HbA1c levels at both 24 weeks and at the end of the study, compared with those receiving placebo. However, no significant differences were observed between the two groups regarding serious adverse events, treatment discontinuation due to adverse events, COVID-19 infections, or URTIs. Plozasiran treatment also did not result in significant changes in platelet count by the end of the study, nor did it meaningfully affect AST or ALT levels at either 24 weeks or at the end of the study.

Subgroup analyses revealed a dose-dependent pattern for certain safety outcomes. The 50 mg quarterly dose was associated with a significant increase in HbA1c, ALT levels, and the overall incidence of adverse events at 24 weeks. The 25 mg quarterly dose led to a significant elevation in AST levels at 24 weeks and ALT levels at both 24 weeks and the end of the study. In contrast, the 10 mg quarterly dose was associated only with a significant increase in AST levels at 24 weeks.For all other safety outcomes, subgroup analyses did not reveal clinically meaningful differences across the various plozasiran doses when compared to placebo.

### Explanation of Findings

To our knowledge, this is the first meta-analysis to focus exclusively on plozasiran, the most recently investigated APOC-III inhibitor. Despite the limited number of included trials, this meta-analysis provides novel insights by synthesizing the totality of evidence on plozasiran’s efficacy and safety. Unlike individual studies, which may be underpowered to detect statistically significant effects across all outcomes or subgroups, our pooled analysis enables more precise effect estimates, clarifies consistency across different dosing regimens, and reveals dose-dependent patterns in both efficacy and adverse effects. Moreover, by comparing findings across diverse populations and trial designs, we offer a broader understanding of plozasiran’s therapeutic potential and safety profile. Plozasiran is a siRNA that specifically targets and degrades mRNA encoding APOC-III. Similar to other APOC-III inhibitors, plozasiran has demonstrated substantial efficacy in improving lipid profiles and shows promise in the treatment of various dyslipidemic disorders.

The breadth of plozasiran’s effects can be better understood by examining the role of its target protein. APOC-III is a glycoprotein synthesized in hepatocytes and found in several lipoproteins, including chylomicrons (CMs), VLDL, LDL, and HDL. It exerts a significant influence on lipoprotein metabolism through both lipoprotein lipase (LPL)–dependent and LPL-independent mechanisms. Notably, APOC-III inhibits LPL activity and disrupts ApoE-mediated hepatic clearance of CMs and VLDL remnants, leading to elevated levels of TRLs.

In hypertriglyceridemia, the liver produces VLDL particles enriched with APOC-III instead of ApoE, which impairs their clearance and promotes the formation of LDL, thereby exacerbating disease progression [24]. Studies have shown that nearly all VLDL particles containing APOC-III undergo intravascular triglyceride lipolysis, generating LDL particles that also contain APOC-III [25–27]. Moreover, VLDL and LDL particles carrying APOC-III—but not those lacking APOC-III—initiate atherosclerosis by promoting endothelial injury and monocyte adhesion [28, 29]. Consequently, APOC-III is considered an independent risk factor for cardiovascular disease [30, 31].

Individuals with loss-of-function mutations in APOC-III exhibit significantly reduced levels of triglycerides and remnant cholesterol, along with a nearly 40% reduction in ASCVD risk [32, 33]. As demonstrated in both RCTs and our meta-analysis, plozasiran’s inhibition of APOC-III synthesis leads to significant improvements in multiple lipid parameters. These findings support its potential utility across a range of dyslipidemic disorders and suggest a role in reducing the risk of conditions such as acute pancreatitis and ASCVD, particularly in patients with hypertriglyceridemia and mixed dyslipidemia.

Current guideline-recommended treatments for severe hypertriglyceridemia include lifestyle modifications—such as dietary changes and increased physical activity—as well as pharmacologic interventions with fibrates and omega-3 fatty acids [34, 35]. However, these approaches have shown limited efficacy in reducing triglyceride levels and preventing acute pancreatitis in clinical trials [8, 36, 37].

In contrast, recent trials by Watts et al. (2025) [17] and Gaudet et al. (2024) [23] demonstrated that plozasiran was able to achieve guideline-recommended triglyceride targets (< 500 mg/dL) and reduce the incidence of acute pancreatitis events [Watts 2025; Gaudet 2024]. Beyond its triglyceride-lowering effects, plozasiran also improves levels of non–HDL cholesterol, HDL-C, and apolipoprotein B (ApoB), further supporting its role in ASCVD risk reduction. This is particularly relevant given the growing body of evidence indicating that non–HDL cholesterol and ApoB are important predictors of ASCVD risk [38, 39].

While reducing LDL-C is widely recognized as beneficial for improving clinical outcomes in patients with ASCVD, plozasiran demonstrated only modest effects on LDL-C—occasionally showing slight increases. This phenomenon is likely attributable to the accelerated conversion of TRLs into LDL particles following APOC-III inhibition. The removal of APOC-III’s suppression of LPL enhances TRL lipolysis, and the concurrent reduction in cholesteryl ester transfer protein (CETP) activity limits the normal exchange of LDL cholesterol for triglycerides in VLDL particles. Consequently, when using potent triglyceride-lowering agents such as plozasiran, it is important to monitor LDL-C levels and consider adjunctive LDL-targeted therapies as needed to achieve comprehensive lipid management [23, 40].

Concerning safety findings for plozasiran, we observed slight increases in HbA1c, AST, and ALT levels, along with a higher incidence of headaches. Subgroup analyses confirmed that changes in HbA1c, ALT, and AST levels were dose- and regimen-dependent.

Ballantyne et al. (2024) and Gaudet et al. (2024) attributed the rise in HbA1c to enhanced LPL activity on TRLs, which may stimulate hepatic gluconeogenesis [16, 23]. However, similar effects have previously been reported with volanesorsen and were not associated with clinically significant deterioration in glycemic control, even after long-term use of up to five years [41, 42]. These findings suggest that glycemic disturbances may be transient and, if necessary, manageable with standard antihyperglycemic therapy [43]. Moreover, the rises seen in liver transaminases were considered non-serious and associated with higher and more frequent doses of plozasiran, which trials mitigated by changing dosing and frequency [16, 22]. As for headache, our pooled analysis of all arms revealed a stark association between plozasiran and headache events, an adverse event reported in 5 or more patients in most included studies [16, 17, 23], which requires more elaboration in future studies.

Although significant heterogeneity was observed in our analysis, several factors likely account for this variability. One key source is the differences in target populations across the included studies, which reflected their varying study objectives. For instance, Gaudet et al. (2023, 2024) enrolled patients with severe hypertriglyceridemia to evaluate plozasiran’s lipid-lowering efficacy [22, 23]. Watts et al. (2025) focused on individuals with genetically confirmed familial chylomicronemia syndrome (FCS) or symptomatic persistent chylomicronemia, aiming to assess reductions in acute pancreatitis events [17]. In contrast, Ballantyne et al. (2024) included patients with mixed dyslipidemia to explore whether plozasiran’s lipid-lowering effects could extend to individuals at risk for ASCVD and support the rationale for a dedicated cardiovascular outcomes trial [16].

A second contributor to heterogeneity was the variation in dosing and administration regimens among studies, as detailed in the summary table Table 1. To address this, we stratified the data based on dose and regimen, which successfully mitigated heterogeneity for several of the measured outcomes.

### Limitations

Our study has several limitations. First, substantial heterogeneity was observed across many outcomes, largely due to variability in study populations, dosing, and administration regimens. Although we addressed this by analyzing outcomes at the 24-week mark and conducting subgroup analyses based on dose and regimen, additional studies focusing on specific patient populations and standardized plozasiran dosing strategies are needed to further validate its efficacy across different dyslipidemic conditions.

Second, while our findings suggest that plozasiran may help reduce ASCVD risk, we were unable to directly assess clinical outcomes such as cardiovascular events, as done with established therapies like statins, fibrates, and omega-3 fatty acids [44, 45], due to the unavailability of such data. Future studies designed to measure clinical endpoints in at-risk populations are essential to confirm plozasiran’s role in ASCVD prevention. Furthermore, direct/indirect comparisons between plozasiran and other lipid-lowering agents are required to further assert plozasiran’s role in dyslipidemia and ASCVD management among other established therapies in real-world clinical settings.

Third, the limited duration of drug administration and relatively short follow-up periods in some included studies may have led to an underestimation of long-term safety risks. Extended follow-up and consistent dosing regimens in future trials will be crucial for accurately characterizing plozasiran’s long-term safety profile.

## Conclusion

In conclusion, our meta-analysis demonstrated that plozasiran significantly improved levels of TG, APOC-III, HDL-C, non–HDL-C, and ApoB. Although slight increases in adverse events, headaches, HbA1c, AST, and ALT were observed, these effects were generally mild, supporting the overall safety profile of plozasiran.

While Plozasiran shows strong potential as a therapeutic option for severe dyslipidemic conditions, further studies are needed to compare its efficacy and safety with currently available treatments and, more importantly, evaluate its impact on clinical outcomes. Such research will be essential to establish plozasiran’s role in routine clinical practice.

## Supplementary Information

Below is the link to the electronic supplementary material.

Supplementary Material 1

Supplementary Material 2

## Data Availability

All data generated or analyzed during this study are included in this published article or the data repositories listed in References.
