# Supplementary material for: The Efficacy and Safety of Plozasiran on Lipid Profile in Dyslipidemic Disorders: A Systematic Review and Meta-Analysis
Source: Cardiovasc Drugs Ther. 2025 Nov 18;40(3):1085–106. doi: 10.1007/s10557-025-07798-8 (PMC13171978; doi:10.1007/s10557-025-07798-8)
Supplement: Supplementary file 1 — Supplementary Material 1 [file 10557_2025_7798_MOESM1_ESM.docx]

**The Efficacy and Safety of Plozasiran on Lipid Profile in Dyslipidemic Disorders: A Systematic Review and Meta-Analysis**

**Search strategy:**

**Pubmed:**

1- (“Apolipoprotein C-3” OR “Apolipoprotein C3” OR “Apolipoprotein C 3” OR “Apolipoprotein C-III” OR “Apolipoprotein CIII” OR “Apolipoprotein CIII” OR ApoC-3 OR ApoC-III OR ApoC3 OR ApoCIII OR “ApoC 3” OR “ApoC III”) AND (inhibit* OR siRNA OR “short-interfer* RNA” OR “small-interfer* RNA” OR “short interfer* RNA” OR “small interfer* RNA” OR “RNA interfer*” OR “antisense oligonucleotide”)

2- (Plozasiran OR ARO-APOC3)

3- #1 OR #2

No limitation were applied.

Field of search: All fields

From inception to February 19, 2025.

Results: **782**

**Web of Science:**

1- ALL= (“Apolipoprotein C-3” OR “Apolipoprotein C3” OR “Apolipoprotein C 3” OR “Apolipoprotein C-III” OR “Apolipoprotein CIII” OR “Apolipoprotein CIII” OR ApoC-3 OR ApoC-III OR ApoC3 OR ApoCIII OR “ApoC 3” OR “ApoC III”) AND (inhibit* OR siRNA OR “short-interfer* RNA” OR “small-interfer* RNA” OR “short interfer* RNA” OR “small interfer* RNA” OR “RNA interfer*” OR “antisense oligonucleotide”)

2- ALL= (Plozasiran OR ARO-APOC3)

3- ALL= (#1 OR #2)

No limitation were applied.

From inception to February 19, 2025.

Results: **832**

**SCOPUS:**

1-TITLE-ABS-KEY (“Apolipoprotein C-3” OR “Apolipoprotein C3” OR “Apolipoprotein C 3” OR “Apolipoprotein C-III” OR “Apolipoprotein CIII” OR “Apolipoprotein CIII” OR ApoC-3 OR ApoC-III OR ApoC3 OR ApoCIII OR “ApoC 3” OR “ApoC III”) AND (inhibit* OR siRNA OR “short-interfer* RNA” OR “small-interfer* RNA” OR “short interfer* RNA” OR “small interfer* RNA” OR “RNA interfer*” OR “antisense oligonucleotide”)

2-TITLE-ABS-KEY (Plozasiran OR ARO-APOC3)

3-TITLE-ABS-KEY (#1 OR #2)

No limitation were applied.

From inception to February 19, 2025.

Results: **1,421**

**CENTRAL:**

1- (“Apolipoprotein C-3” OR “Apolipoprotein C3” OR “Apolipoprotein C 3” OR “Apolipoprotein C-III” OR “Apolipoprotein CIII” OR “Apolipoprotein CIII” OR ApoC-3 OR ApoC-III OR ApoC3 OR ApoCIII OR “ApoC 3” OR “ApoC III”) AND (inhibit* OR siRNA OR “short-interfer* RNA” OR “small-interfer* RNA” OR “short interfer* RNA” OR “small interfer* RNA” OR “RNA interfer*” OR “antisense oligonucleotide”)

2- (Plozasiran OR ARO-APOC3)

3- #1 OR #2

No limitation were applied.

Field of search: All fields

From inception to February 19, 2025.

Results: **153**

**Supplementary figures:**


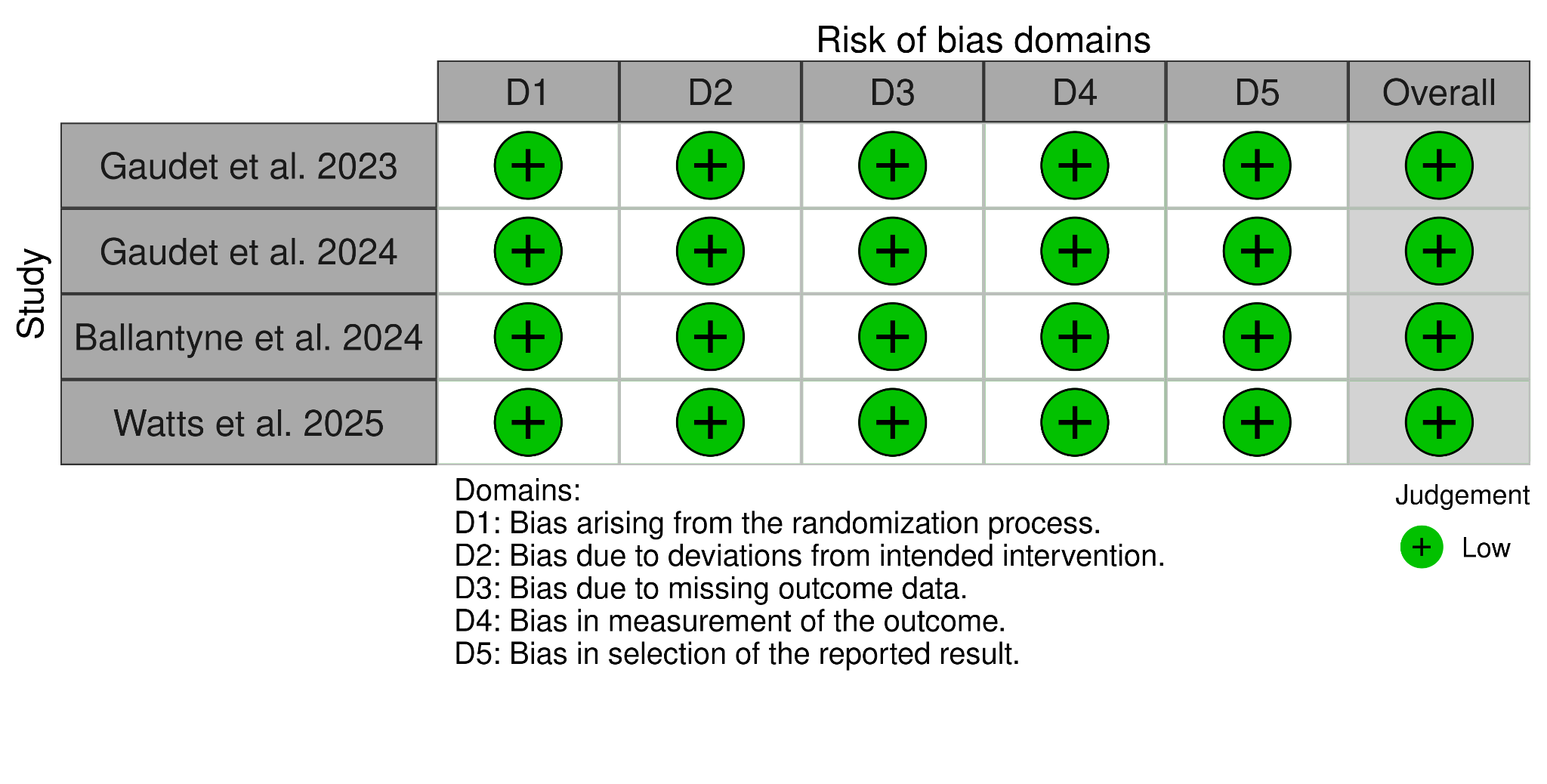


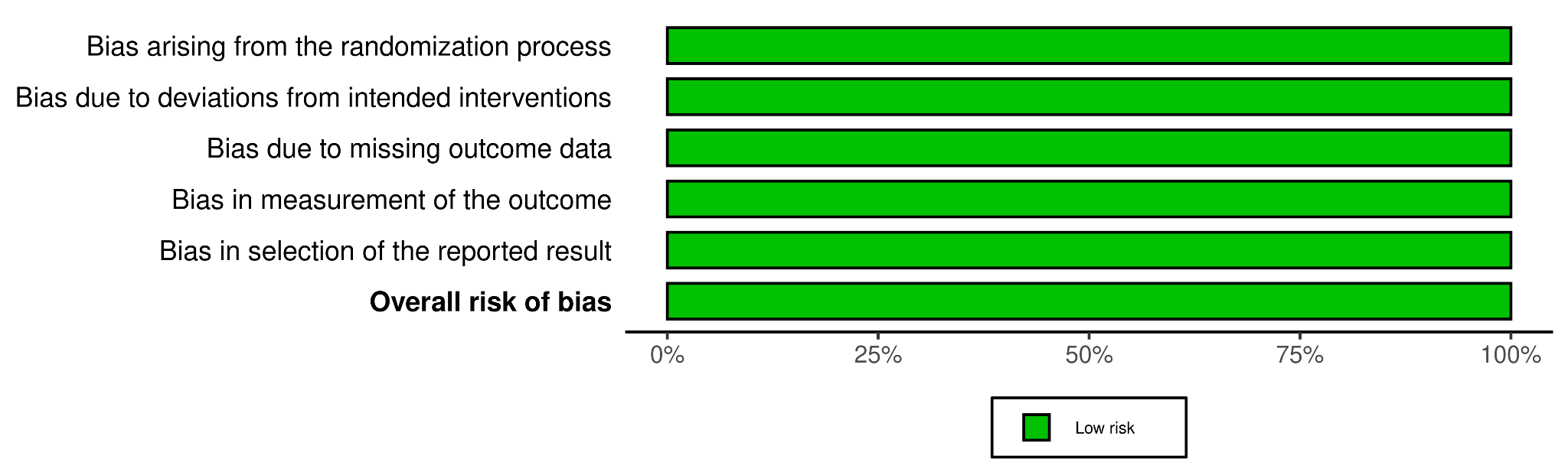


Supplementary Figure 1: Risk of bias assessment of RCTs.


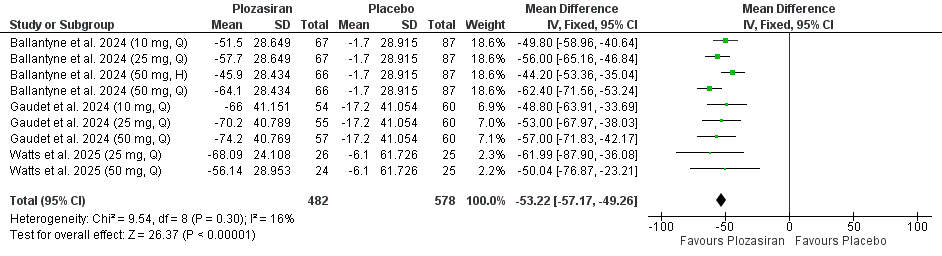


Supplementary Figure 2A: Forrest plot demonstrating percent change from baseline in TG levels at 24 weeks.


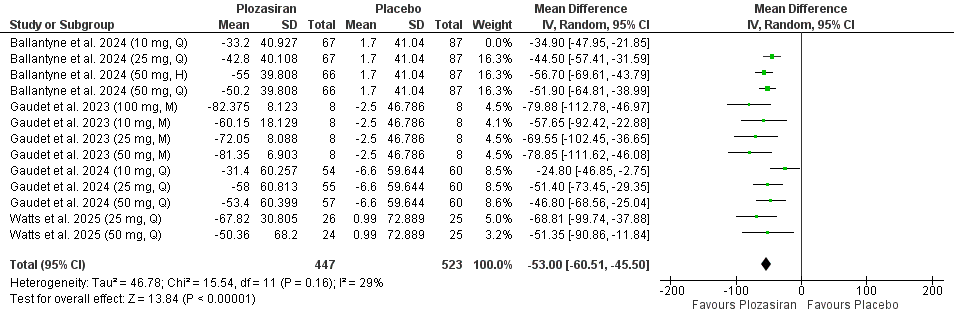


Supplementary Figure 2B: Forrest plot demonstrating percent change from baseline in TG levels at the end of the study.


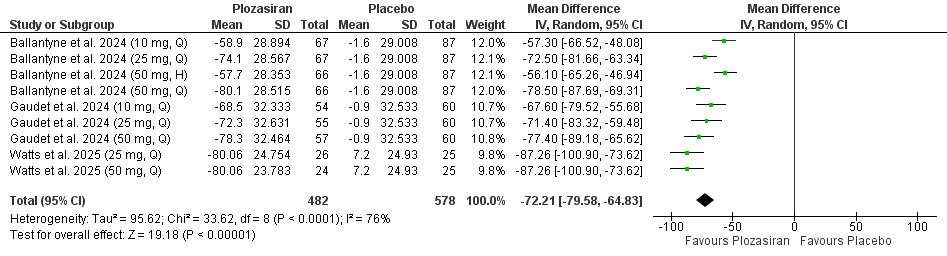


Supplementary Figure 3A: Forrest plot demonstrating percent change from baseline in APOC-III levels at 24 weeks.


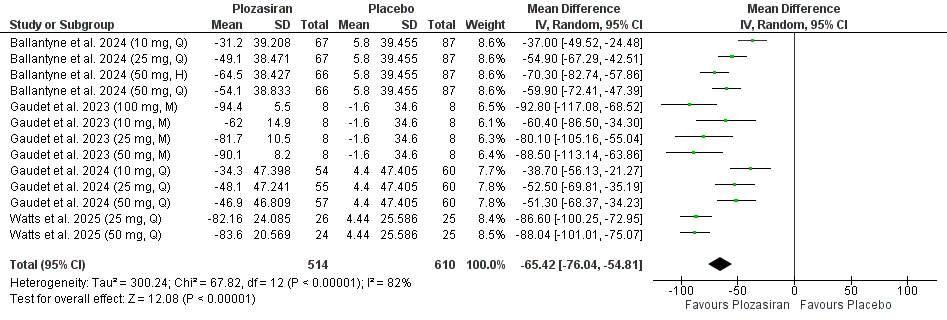


Supplementary Figure 3B: Forrest plot demonstrating percent change from baseline in APOC-III levels at the end of the study.


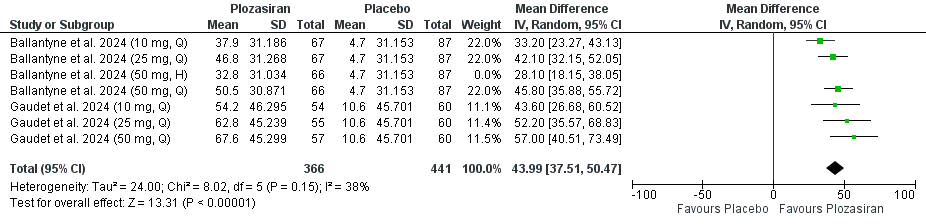


Supplementary Figure 4A: Forrest plot demonstrating percent change from baseline in HDL-C levels at 24 weeks.


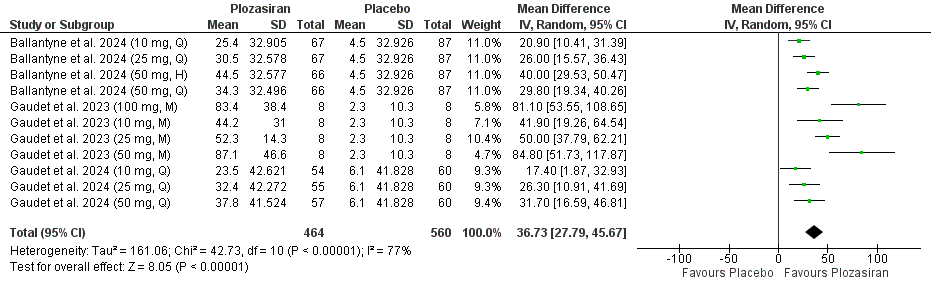


Supplementary Figure 4B: Forrest plot demonstrating percent change from baseline in HDL-C levels at the end of the study.


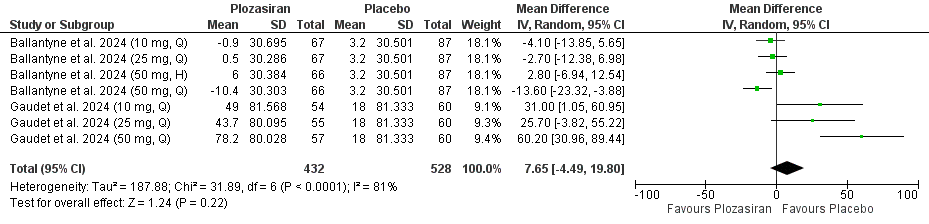


Supplementary Figure 5A: Forrest plot demonstrating percent change from baseline in LDL-C levels at 24 weeks.


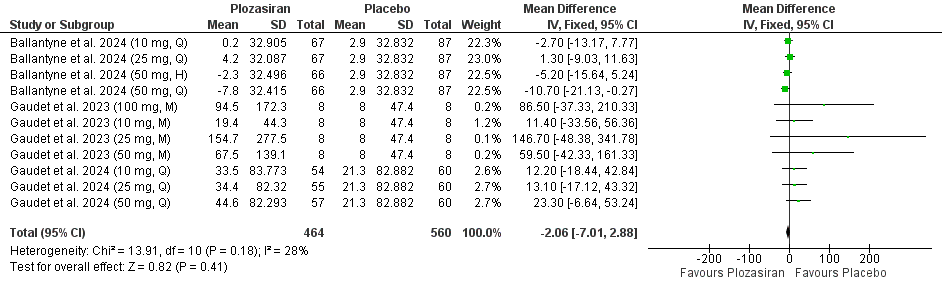


Supplementary Figure 5B: Forrest plot demonstrating percent change from baseline in LDL-C levels at the end of the study.


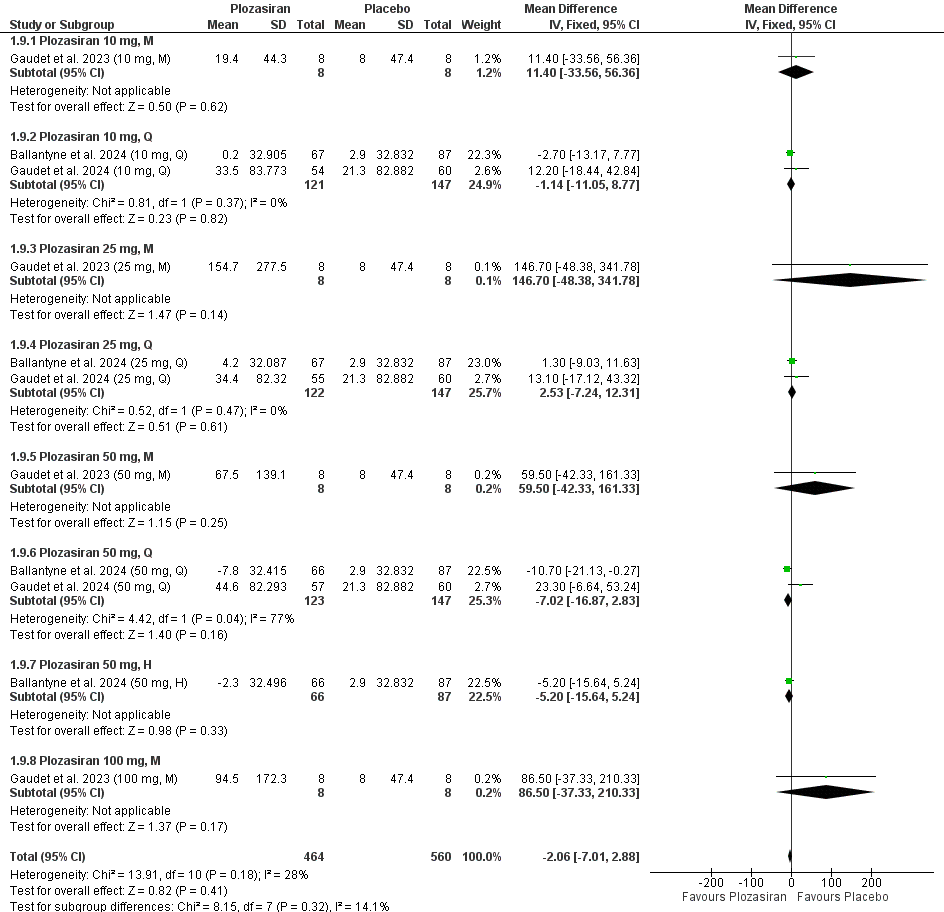


Supplementary Figure 5C: Forrest plot demonstrating percent change from baseline in LDL-C levels at the end of the study (subgroup analysis).


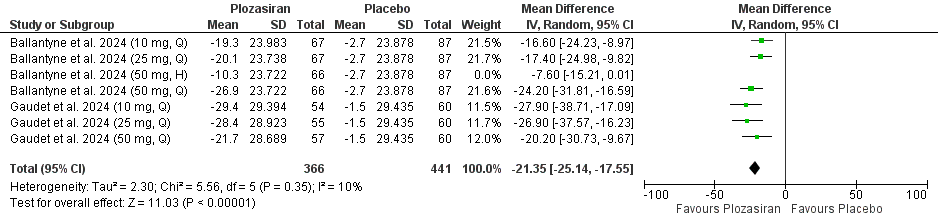


Supplementary Figure 6A: Forrest plot demonstrating percent change from baseline in Non-HDL cholesterol levels at 24 weeks.


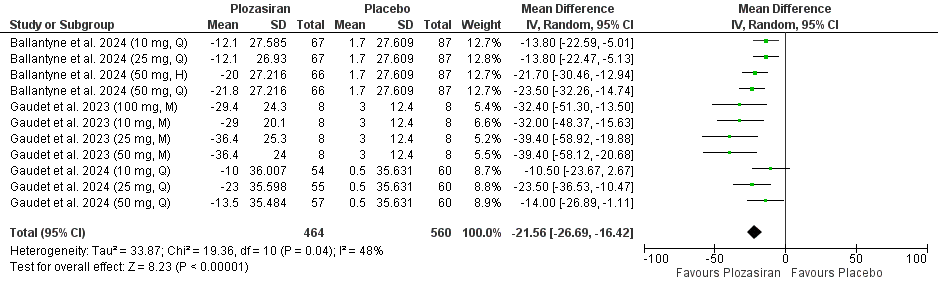


Supplementary Figure 6B: Forrest plot demonstrating percent change from baseline in Non-HDL cholesterol levels at the end of the study.


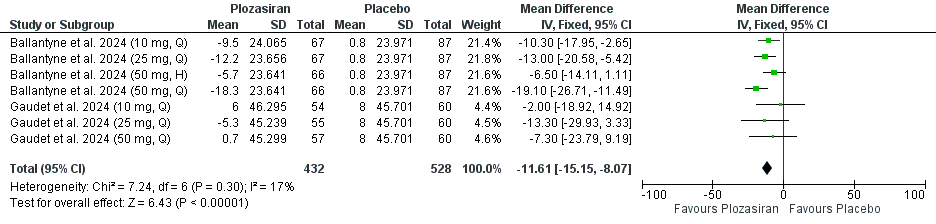


Supplementary Figure 7A: Forrest plot demonstrating percent change from baseline in ApoB levels at 24 weeks.


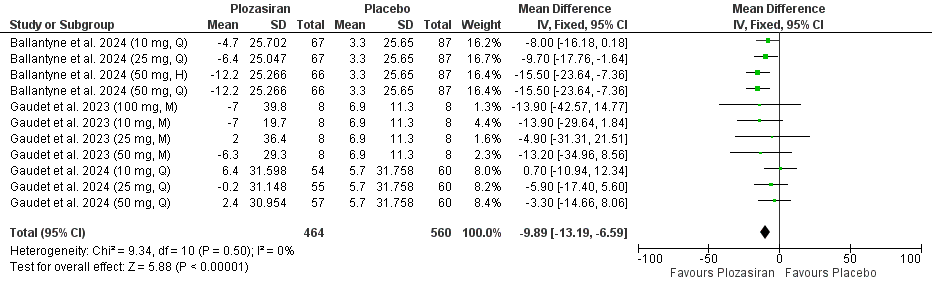


Supplementary Figure 7B: Forrest plot demonstrating percent change from baseline in ApoB levels at the end of the study.


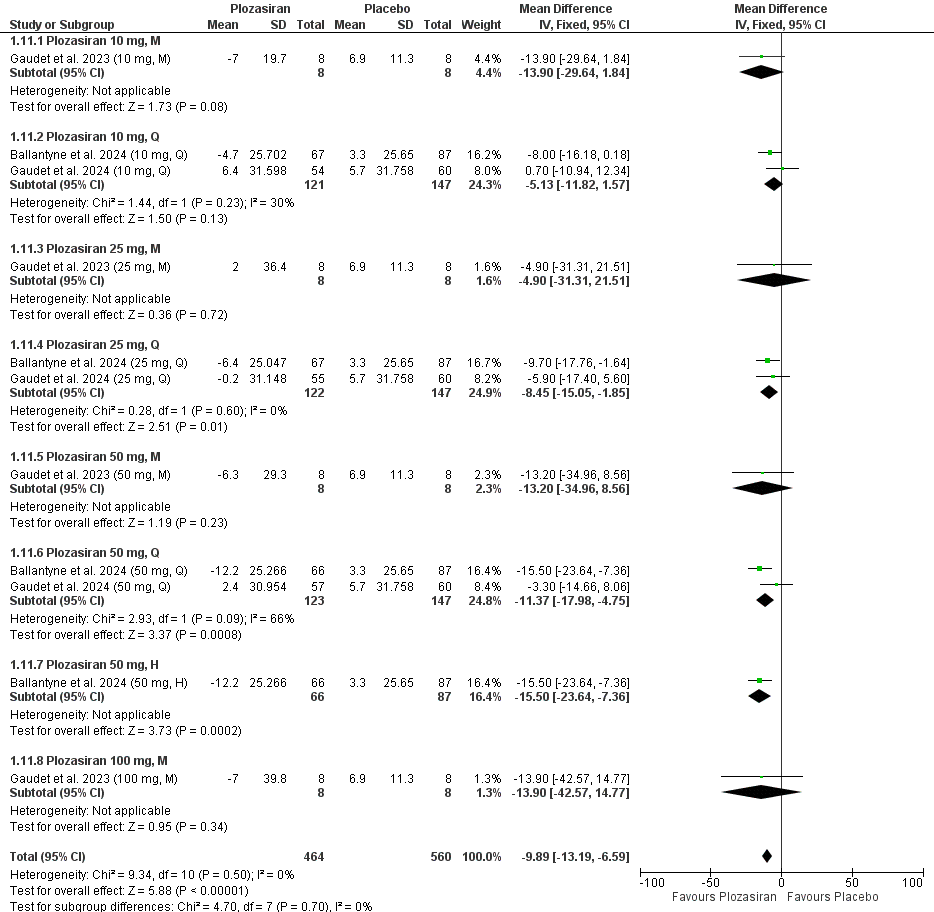


Supplementary Figure 7C: Forrest plot demonstrating percent change from baseline in ApoB levels at the end of the study (subgroup analysis).


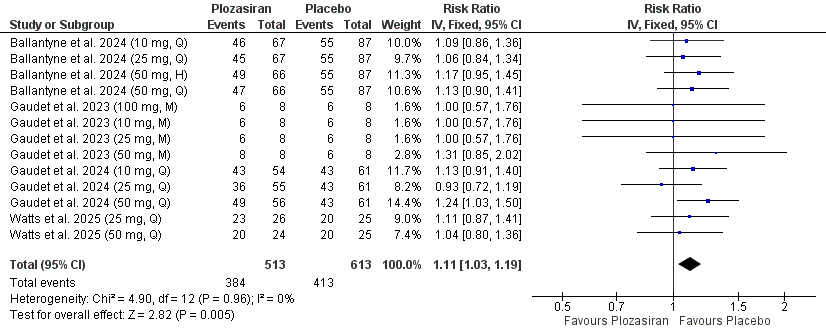


Supplementary Figure 8: Forrest plot demonstrating Any adverse events at the end of the study.


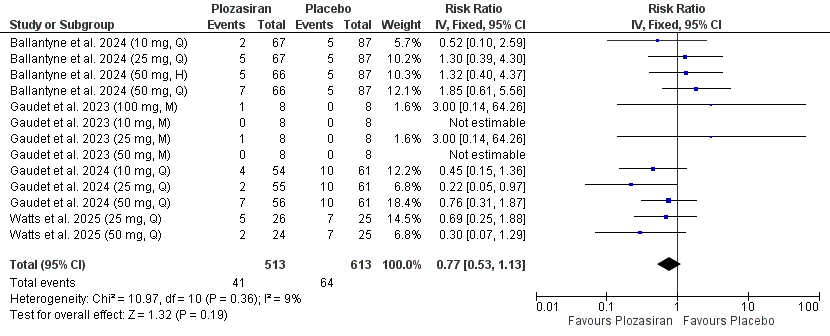


Supplementary Figure 9: Forrest plot demonstrating Serious adverse events at the end of the study.


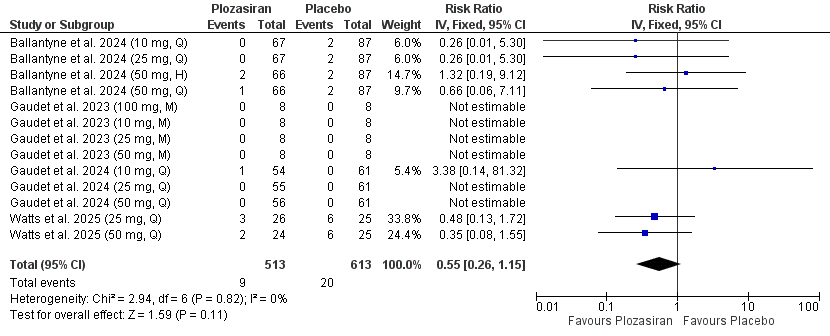


Supplementary Figure 10: Forrest plot demonstrating adverse events leading to discontinuation of the drug at the end of the study.


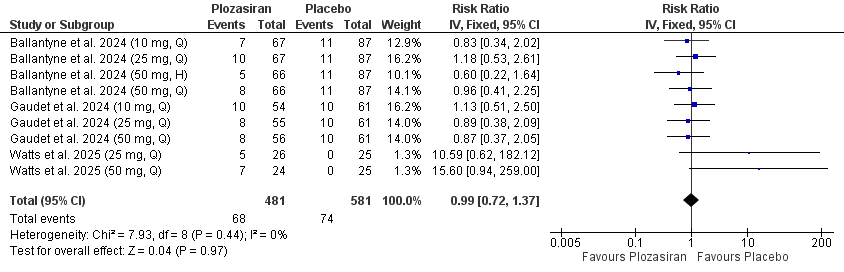


Supplementary Figure 11: Forrest plot demonstrating Covid-19 at the end of the study.


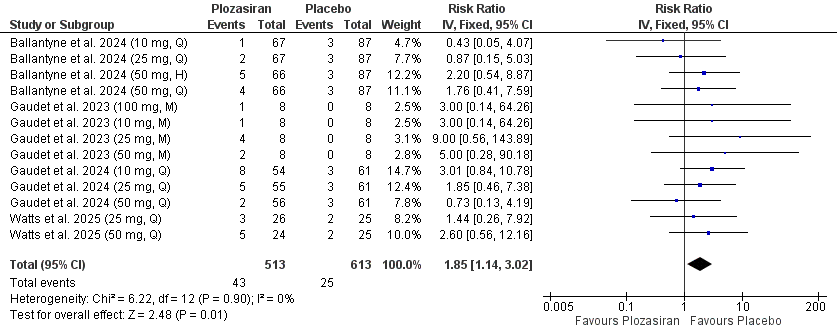


Supplementary Figure 12: Forrest plot demonstrating Headache at the end of the study.


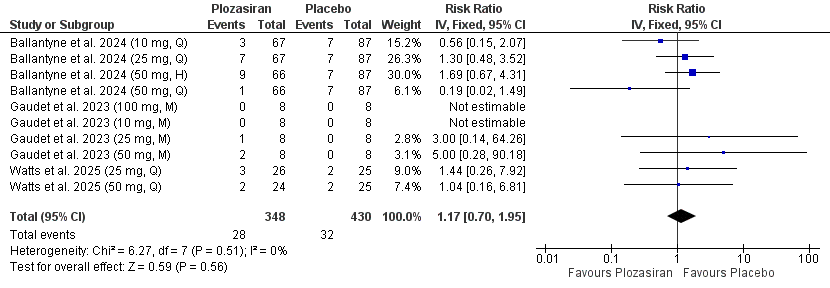


Supplementary Figure 13: Forrest plot demonstrating URTI at the end of the study.


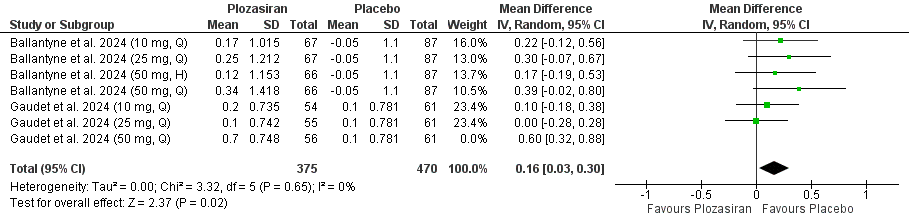


Supplementary Figure 14A: Forrest plot demonstrating Absolute change from baseline in HbA1C at 24 weeks.


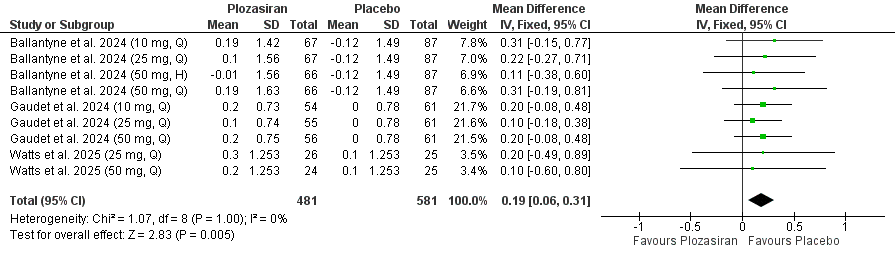


Supplementary Figure 14B: Forrest plot demonstrating Absolute change from baseline in HbA1C at the end of the study.


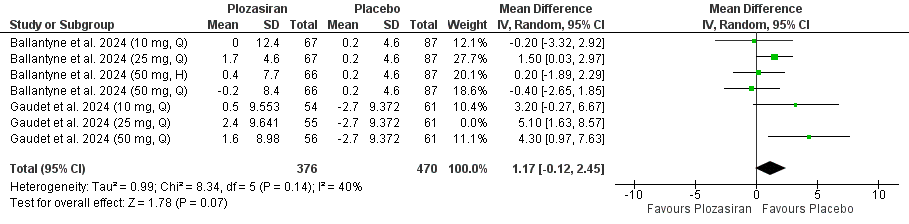


Supplementary Figure 15A: Forrest plot demonstrating Absolute change from baseline in AST at 24 weeks.


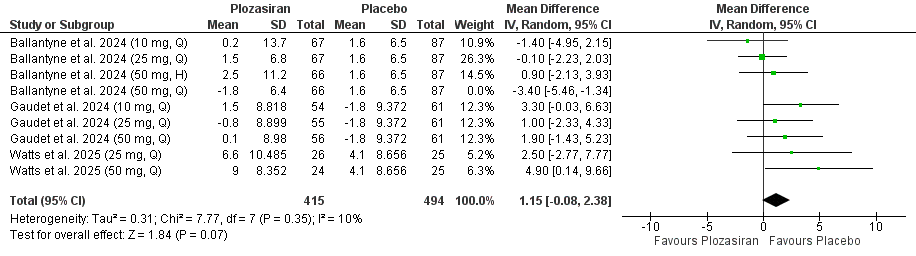


Supplementary Figure 15B: Forrest plot demonstrating Absolute change from baseline in AST at the end of the study.


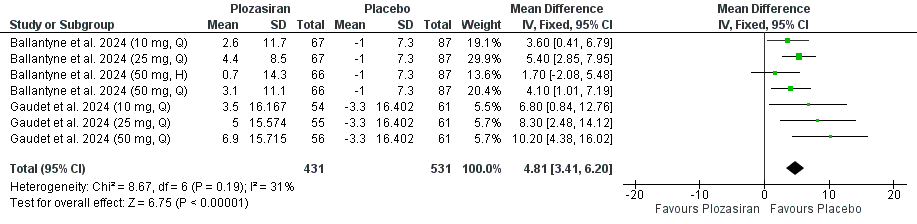


Supplementary Figure 16A: Forrest plot demonstrating Absolute change from baseline in ALT at 24 weeks.


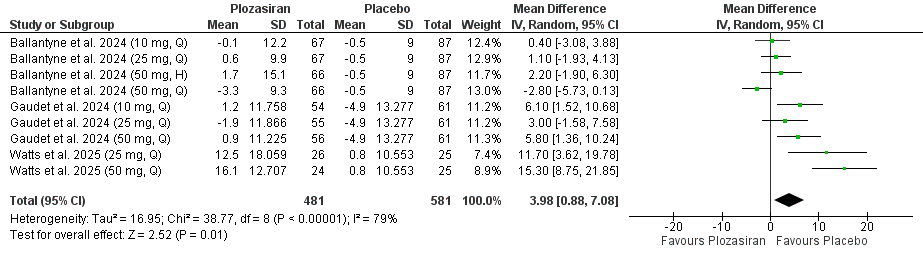


Supplementary Figure 16B: Forrest plot demonstrating Absolute change from baseline in ALT at the end of the study.


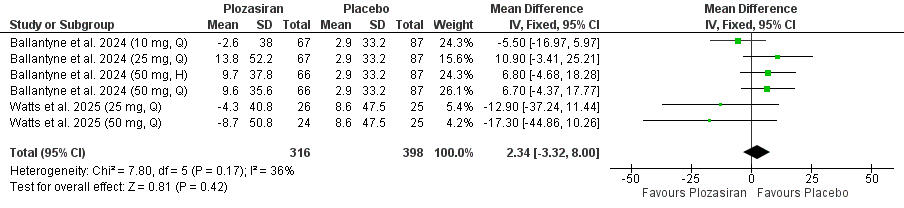


Supplementary Figure 17: Forrest plot demonstrating Absolute change in platelets count at the end of the study.
