# Supplementary material for: The Efficacy and Safety of Plozasiran on Lipid Profile in Dyslipidemic Disorders: A Systematic Review and Meta-Analysis
Source: Cardiovasc Drugs Ther. 2025 Nov 18;40(3):1085–106. doi: 10.1007/s10557-025-07798-8 (PMC13171978; doi:10.1007/s10557-025-07798-8)
Supplement: Supplementary file 2 — Supplementary Material 2 [file 10557_2025_7798_MOESM2_ESM.docx]

**Subgroup Analysis**

**Efficacy Outcomes**

**Percent change from baseline in TG level**

Our subgroup analysis at 24 weeks, stratified by regimen and doses of **Plozasiran**, compared with placebo, revealed statistically significant reductions in triglyceride (TG) levels. Specifically, the reductions were observed in the following groups: 10 mg Q (MD = -49.53, 95% CI [-57.37, -41.70], P < 0.00001), 25 mg Q (MD = -55.75, 95% CI [-63.23, -48.27], P < 0.00001), 50 mg Q (MD = -60.06, 95% CI [-67.55, -52.58], P < 0.00001), and 50 mg H (MD = -44.20, 95% CI [-53.36, -35.04], P < 0.00001). The 50mg H group was based on single-study data.

Homogeneity was demonstrated in the 10 mg Q (P = 0.91, I² = 0%), 25 mg Q (P = 0.84, I² = 0%), and 50 mg Q (P = 0.62, I² = 0%) groups. However, homogeneity could not be assessed in the 50 mg H group, as the outcome was reported in only one study. **(Supplementary file Fig 18)**

**Percent change from baseline in APOC-III level**

Our subgroup analysis at 24 weeks, stratified by regimen and doses of **Plozasiran**, compared with placebo, demonstrated statistically significant reductions in APOC-III levels. Specifically, reductions were observed in the following groups: 10 mg Q (MD = -61.16, 95% CI [-68.45, -53.86], P < 0.00001), 25 mg Q (MD = -75.44, 95% CI [-81.86, -69.03], P < 0.00001), 50 mg Q (MD = -80.10, 95% CI [-86.50, -73.70], P < 0.00001), and 50 mg H (MD = -56.10, 95% CI [-65.26, -46.94], P < 0.00001). The 50mg H group was based on single-study data.

Homogeneity was observed in the 10 mg Q (P = 0.18, I² = 44%), 25 mg Q (P = 0.16, I² = 46%), and 50 mg Q (P = 0.50, I² = 0%) groups. However, homogeneity could not be assessed in the 50 mg H group, as the outcome was reported in only one study. **(Supplementary file Fig 19)**

**Percent change from baseline in HDL-C level**

Our subgroup analysis at 24 weeks, stratified by regimen and doses of **Plozasiran**, compared with placebo, revealed statistically significant increases in HDL-C levels. Specifically, the following groups showed significant improvements: 10 mg Q (MD = 35.87, 95% CI [27.30, 44.43], P < 0.00001), 25 mg Q (MD = 44.76, 95% CI [36.22, 53.30], P < 0.00001), 50 mg Q (MD = 48.77, 95% CI [40.28, 57.27], P < 0.00001), and 50 mg H (MD = 28.10, 95% CI [18.15, 38.05], P < 0.00001). The 50mg H group was based on single-study data.

Homogeneity was observed in the 10 mg Q (P = 0.30, I² = 7%), 25 mg Q (P = 0.31, I² = 4%), and 50 mg Q (P = 0.25, I² = 23%) groups. However, homogeneity could not be assessed in the 50 mg H group, as the outcome was reported in only one study. **(Supplementary file Fig 20)**

**Percent change from baseline in LDL-C level**

Our subgroup analysis at 24 weeks, stratified by regimen and doses of **Plozasiran**, compared with placebo, showed no statistically significant differences in LDL-C levels. Specifically, there were no significant changes in the following groups: 10 mg Q (MD = -0.73, 95% CI [-10.01, 8.54], P = 0.88), 25 mg Q (MD = 0.06, 95% CI [-9.14, 9.25], P = 0.99), 50 mg Q (MD = -6.25, 95% CI [-15.48, 2.97], P = 0.18), and 50 mg H (MD = 2.80, 95% CI [-6.94, 12.54], P = 0.57). The 50mg H group was based on single-study data.

Heterogeneity was observed in the 10 mg Q (P = 0.03, I² = 79%), 25 mg Q (P = 0.07, I² = 69%), and 50 mg Q (P < 0.00001, I² = 95%) groups. However, heterogeneity could not be assessed in the 50 mg H group, as the outcome was reported in only one study. **(Supplementary file Fig 21)**

**Percent change from baseline in Non-HDL cholesterol level**

Our subgroup analysis at 24 weeks, stratified by regimen and doses of **Plozasiran**, compared with placebo, revealed statistically significant reductions in Non-HDL cholesterol levels. Specifically, reductions were observed in the 10 mg Q (MD = -20.35, 95% CI [-26.59, -14.12], P < 0.00001), 25 mg Q (MD = -20.59, 95% CI [-26.77, -14.41], P < 0.00001), and 50 mg Q (MD = -22.83, 95% CI [-29.00, -16.66], P < 0.00001) groups. However, no statistically significant difference was found in the 50 mg H group (MD = -7.60, 95% CI [-15.21, 0.01], P = 0.05). The 50mg H group was based on single-study data.

Analysis of heterogeneity revealed significant variability in the 10 mg Q group (P = 0.09, I² = 64%). In contrast, homogeneity was observed in the 25 mg Q (P = 0.15, I² = 50%) and 50 mg Q (P = 0.55, I² = 0%) groups. It is important to note that heterogeneity could not be assessed in the 50 mg H group, as the outcome was reported in only one study. **(Supplementary file Fig 22)**

**Percent change from baseline in ApoB level**

Our subgroup analysis at 24 weeks, stratified by regimen and doses of **Plozasiran**, compared with placebo, demonstrated statistically significant reductions in ApoB levels. Specifically, reductions were observed in the 10 mg Q (MD = -8.89, 95% CI [-15.86, -1.92], P = 0.01), 25 mg Q (MD = -13.05, 95% CI [-19.95, -6.15], P = 0.0002), and 50 mg Q (MD = -17.03, 95% CI [-23.94, -10.12], P < 0.00001) groups. However, no statistically significant difference was observed in the 50 mg H group (MD = -6.50, 95% CI [-14.11, 1.11], P = 0.09). The 50mg H group was based on single-study data.

Heterogeneity analysis revealed homogeneity in the 10 mg Q (P = 0.38, I² = 0%), 25 mg Q (P = 0.97, I² = 0%), and 50 mg Q (P = 0.20, I² = 38%) groups. However, this analysis was not applicable in the 50 mg H group, as the outcome was reported in only one study. **(Supplementary file Fig 23)**

**Safety Outcomes**

**Adverse events leading to discontinuation of the drug**

Our subgroup analysis at the end of the study, stratified by regimen and doses of **Plozasiran** compared with placebo, revealed no statistically significant difference in adverse events leading to discontinuation of the drug. Specifically, the 10 mg Q group (RR = 0.88, 95% CI [0.10, 7.82], P = 0.91), 25 mg Q group (RR = 0.44, 95% CI [0.14, 1.41], P = 0.17), 50 mg Q group (RR = 0.42, 95% CI [0.12, 1.48], P = 0.18), and 50 mg H group (RR = 1.32, 95% CI [0.19, 9.12], P = 0.78) all showed no significant differences compared to placebo. The 50mg H group was based on single-study data.

Heterogeneity analysis demonstrated homogeneity in the 10 mg Q (P = 0.25, I² = 24%), 25 mg Q (P = 0.71, I² = 0%), and 50 mg Q (P = 0.66, I² = 0%) groups. However, this analysis was not applicable to the 50 mg H group, as the outcome was reported in only one study. **(Supplementary file Fig 24)**

**Covid-19**

Our subgroup analysis at the end of the study, stratified by regimen and doses of **Plozasiran** compared with placebo, showed no statistically significant difference in COVID-19 infection rates. Specifically, the 10 mg Q group (RR = 0.98, 95% CI [0.54, 1.78], P = 0.96), 25 mg Q group (RR = 1.14, 95% CI [0.64, 2.01], P = 0.66), 50 mg Q group (RR = 1.04, 95% CI [0.57, 1.87], P = 0.91), and 50 mg H group (RR = 0.60, 95% CI [0.22, 1.64], P = 0.32) all demonstrated no significant differences compared to placebo. The 50mg H group was based on single-study data.

Heterogeneity analysis revealed homogeneity in the 10 mg Q group (P = 0.61, I² = 0%), 25 mg Q group (P = 0.26, I² = 26%), and 50 mg Q group (P = 0.15, I² = 47%). However, this analysis was not applicable to the 50 mg H group, as the outcome was reported in only one study. **(Supplementary file Fig 25)**

**Headache**

Our subgroup analysis at the end of the study, stratified by regimen and doses of **Plozasiran**, compared with placebo, revealed no statistically significant difference in the incidence of headache. Specifically, the 10 mg M group (RR = 3.00, 95% CI [0.14, 64.26], P = 0.48), 10 mg Q group (RR = 1.87, 95% CI [0.62, 5.68], P = 0.27), 25 mg M group (RR = 9.00, 95% CI [0.56, 143.89], P = 0.12), 25 mg Q group (RR = 1.40, 95% CI [0.56, 3.50], P = 0.47), 50 mg M group (RR = 5.00, 95% CI [0.28, 90.18], P = 0.28), 50 mg Q group (RR = 1.59, 95% CI [0.64, 3.94], P = 0.32), 50 mg H group (RR = 2.20, 95% CI [0.54, 8.87], P = 0.27), and 100 mg M group (RR = 3.00, 95% CI [0.14, 64.26], P = 0.48) all showed no significant differences compared to placebo. The 10 mg M, 25 mg M, 50 mg M, 50 mg H, and 100 mg M groups were based on single-study data.

Analysis of homogeneity revealed consistent findings in the 10 mg Q group (P = 0.14, I² = 54%), 25 mg Q group (P = 0.80, I² = 0%), and 50 mg Q group (P = 0.55, I² = 0%). However, homogeneity analysis was not applicable to the 10 mg M, 25 mg M, 50 mg M, 50 mg H, and 100 mg M groups, as these outcomes were reported in only one study. **(Supplementary file Fig 26)**

**Upper Respiratory Tract Infections**

Our subgroup analysis at the end of the study, stratified by regimen and doses of **Plozasiran**, compared with placebo, revealed no statistically significant difference in the incidence of **URTI**. Specifically, the 10 mg Q group (RR = 0.56, 95% CI [0.15, 2.07], P = 0.38), 25 mg M group (RR = 3.00, 95% CI [0.14, 64.26], P = 0.48), 25 mg Q group (RR = 1.33, 95% CI [0.56, 3.16], P = 0.51), 50 mg M group (RR = 5.00, 95% CI [0.28, 90.18], P = 0.28), 50 mg Q group (RR = 0.48, 95% CI [0.12, 1.93], P = 0.30), and 50 mg H group (RR = 1.69, 95% CI [0.67, 4.31], P = 0.27) showed no significant differences compared to placebo. The 10 mg M, 25 mg M, 50 mg M, 50 mg H, and 100 mg M groups were based on single-study data.

Homogeneity analysis demonstrated consistency in the 25 mg Q group (P = 0.92, I² = 0%) and the 50 mg Q group (P = 0.23, I² = 30%). However, homogeneity analysis was not applicable to the 10 mg M, 25 mg M, 50 mg M, 50 mg H, and 100 mg M groups, as these outcomes were reported in only one study. **(Supplementary file Fig 27)**

**Absolute change from baseline in HbA1C level**

Our subgroup analysis at 24 weeks, stratified by regimen and doses of **Plozasiran**, compared with placebo, revealed no statistically significant differences in **HbA1C** levels for the 10 mg Q group (MD = 0.15, 95% CI [-0.06, 0.36], P = 0.17), 25 mg Q group (MD = 0.11, 95% CI [-0.11, 0.33], P = 0.34), and 50 mg H group (MD = 0.17, 95% CI [-0.19, 0.53], P = 0.36). However, a statistically significant increase in **HbA1C** was observed in the **Plozasiran** arm compared to placebo in the 50 mg Q group (MD = 0.53, 95% CI [0.30, 0.76], P < 0.00001). The 50mg H group was based on single-study data.

Homogeneity analysis demonstrated consistency in the 10 mg Q group (P = 0.59, I² = 0%), 25 mg Q group (P = 0.20, I² = 38%), and 50 mg Q group (P = 0.41, I² = 0%). However, homogeneity analysis was not applicable to the 50 mg H group, as this outcome was reported in only one study. **(Supplementary file Fig 28A)**

At the end of the study, our subgroup analysis, stratified by regimen and doses of **Plozasiran**, compared with placebo, showed no statistically significant differences in **HbA1C** levels for the 10 mg Q group (MD = 0.23, 95% CI [-0.01, 0.47], P = 0.06), 25 mg Q group (MD = 0.14, 95% CI [-0.09, 0.36], P = 0.24), 50 mg Q group (MD = 0.21, 95% CI [-0.02, 0.44], P = 0.07), and 50 mg H group (MD = 0.11, 95% CI [-0.38, 0.60], P = 0.66). The 50mg H group was based on single-study data.

Homogeneity analysis demonstrated consistency in the 10 mg Q group (P = 0.69, I² = 0%), 25 mg Q group (P = 0.90, I² = 0%), and 50 mg Q group (P = 0.88, I² = 0%). Again, homogeneity analysis was not applicable to the 50 mg H group, as the outcome was reported in only one study. **(Supplementary file Fig 28B)**

**Absolute change from baseline in AST level**

Our subgroup analysis at 24 weeks, stratified by regimen and doses of **Plozasiran**, compared with placebo, showed no statistically significant differences in **AST** levels for the 10 mg Q group (MD = 1.32, 95% CI [-1.00, 3.64], P = 0.26), 50 mg Q group (MD = 1.07, 95% CI [-0.79, 2.93], P = 0.26), and 50 mg H group (MD = 0.20, 95% CI [-1.89, 2.29], P = 0.85). However, a statistically significant increase in **AST** levels was observed in the **Plozasiran** arm compared to placebo in the 25 mg Q group (MD = 2.05, 95% CI [0.70, 3.40], P = 0.003). The 50mg H group was based on single-study data.

Homogeneity analysis revealed consistency in the 10 mg Q group (P = 0.59, I² = 0%), 25 mg Q group (P = 0.20, I² = 38%), and 50 mg Q group (P = 0.41, I² = 0%). However, homogeneity analysis was not applicable to the 50 mg H group, as the outcome was reported in only one study. **(Supplementary file Fig 29A)**

At the end of the study, our subgroup analysis, stratified by regimen and doses of **Plozasiran**, compared with placebo, again showed no statistically significant differences in **AST** levels for the 10 mg Q group (MD = 1.11, 95% CI [-1.32, 3.53], P = 0.37), 25 mg Q group (MD = 0.46, 95% CI [-1.24, 2.15], P = 0.60), 50 mg Q group (MD = -1.11, 95% CI [-2.76, 0.53], P = 0.18), and 50 mg H group (MD = 0.90, 95% CI [-2.13, 3.93], P = 0.56). The 50mg H group was based on single-study data.

Further analyses demonstrated heterogeneity in the 10 mg Q group (P = 0.06, I² = 72%) and 50 mg Q group (P = 0.0009, I² = 86%). However, homogeneity was observed in the 25 mg Q group (P = 0.62, I² = 0%). As with earlier findings, homogeneity analysis was not applicable to the 50 mg H group, as the outcome was reported in only one study. **(Supplementary file Fig 29B)**

**Absolute change from baseline in ALT level**

showed statistically significant increases in **ALT** levels in the 10 mg Q group (MD = 4.31, 95% CI [1.50, 7.13], P = 0.003), the 25 mg Q group (MD = 5.87, 95% CI [3.53, 8.20], P <0.00001), and the 50 mg Q group (MD = 5.44, 95% CI [2.71, 8.17], P <0.0001). However, no statistically significant difference in **ALT** levels was observed in the 50 mg H group (MD = 1.70, 95% CI [-2.08, 5.48], P = 0.38) when compared to placebo. The 50mg H group was based on single-study data.

Analysis of homogeneity revealed consistency in the 10 mg Q group (P = 0.35, I² = 0%) and the 25 mg Q group (P = 0.37, I² = 0%). In contrast, heterogeneity was observed in the 50 mg Q group (P = 0.07, I² = 70%). Notably, homogeneity analysis was not applicable to the 50 mg H group, as the outcome was reported in only one study. **(Supplementary file Fig 30A).**

At the study’s conclusion, our subgroup analysis, stratified by regimen and doses of **Plozasiran**, compared with placebo, showed no statistically significant differences in **ALT** levels for the 10 mg Q group (MD = 2.49, 95% CI [-0.28, 5.26], P = 0.08), the 50 mg Q group (MD = 1.71, 95% CI [-0.59, 4.00], P = 0.14), and the 50 mg H group (MD = 2.20, 95% CI [-1.90, 6.30], P = 0.29). However, a statistically significant increase in **ALT** levels was observed in the 25 mg Q group (MD = 2.57, 95% CI [0.16, 4.99], P = 0.04) compared to placebo. The 50mg H group was based on single-study data.

Further analysis revealed heterogeneity in the 10 mg Q group (P = 0.05, I² = 74%), the 25 mg Q group (P = 0.05, I² = 66%), and the 50 mg Q group (P <0.0001, I² = 93%). Homogeneity analysis was not applicable in the 50 mg H group, as the outcome was reported in only one study. **(Supplementary file Fig 30B).**

**Absolute change from baseline in Platelets count**

Our subgroup analysis at the end of the study, stratified by regimen and doses of **Plozasiran**, compared with placebo, showed no statistically significant difference in **platelet count** across the 10 mg Q (MD = -5.50, 95% CI [-16.97, -5.97], P = 0.35), 25 mg Q (MD = 4.79, 95% CI [-7.55, 17.22], P = 0.45), 50 mg Q (MD = 3.37, 95% CI [-6.90, 13.64], P = 0.52), and 50 mg H (MD = 6.80, 95% CI [-4.68, 18.28], P = 0.25) groups. The 10mg Q, 50mg H groups were based on single-study data.

Analysis revealed homogeneity in the 25 mg Q (P = 0.10, I² = 63%) and 50 mg Q (P = 0.11, I² = 60%) groups. However, homogeneity could not be assessed for the 10 mg Q and 50 mg H groups, as the outcome was reported in only one study. **(Supplementary file Fig 31).**


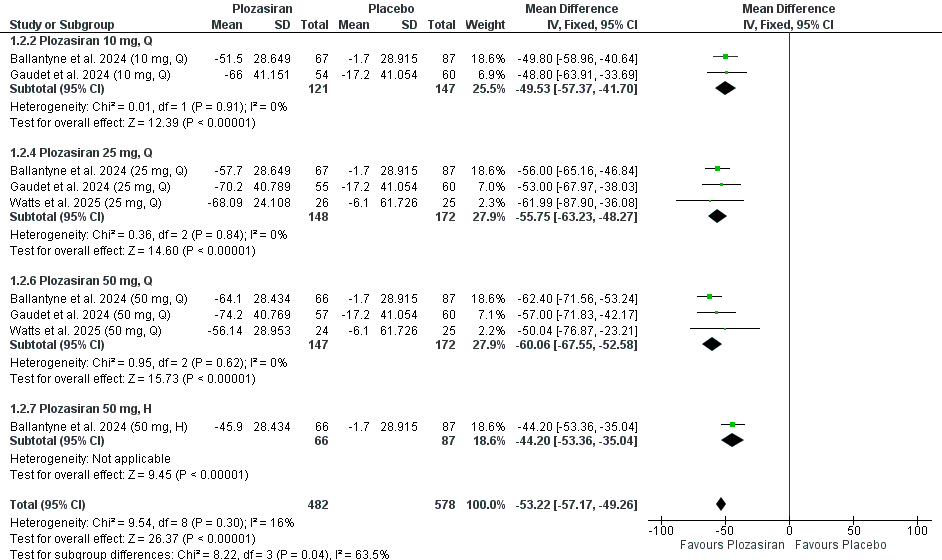


Supplementary Figure 18: Forrest plot demonstrating percent change from baseline in TG levels at 24 weeks (subgroup analysis).


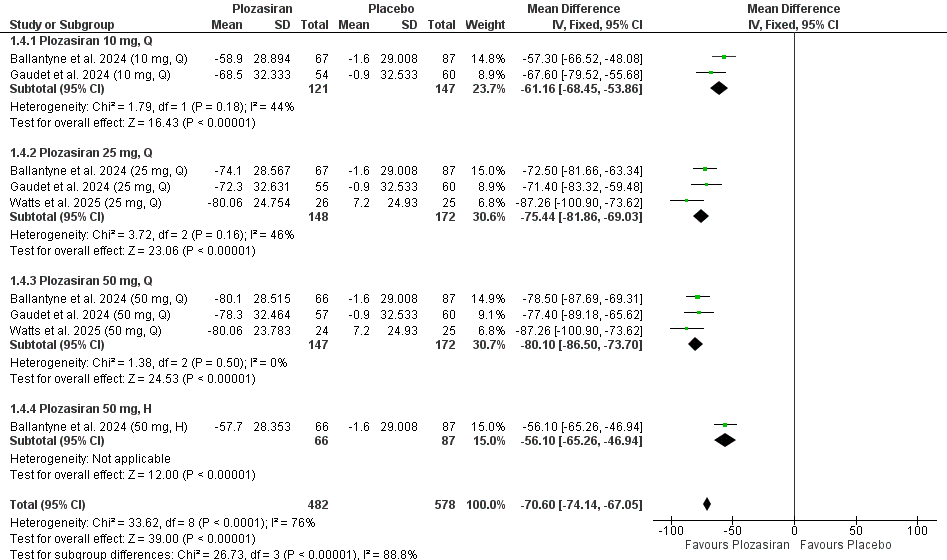


Supplementary Figure 19: Forrest plot demonstrating percent change from baseline in APOC-III levels at 24 weeks (subgroup analysis).


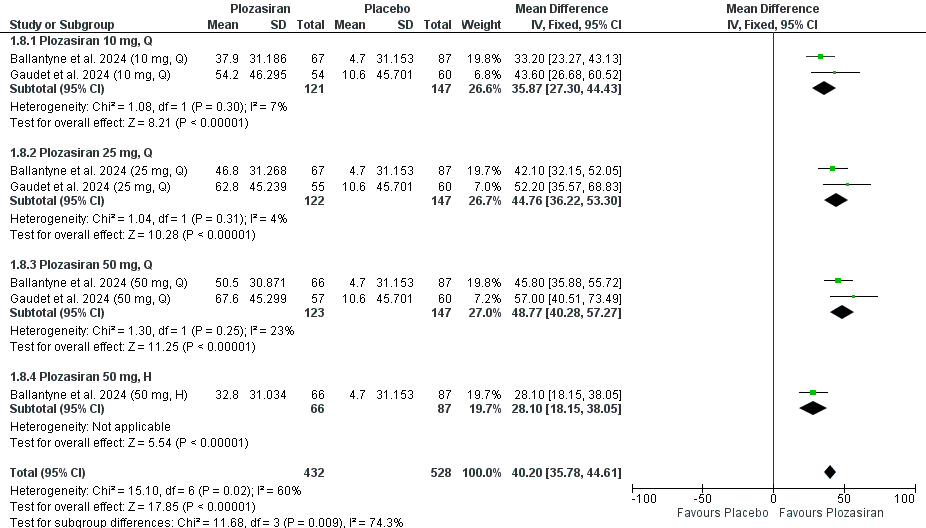


Supplementary Figure 20: Forrest plot demonstrating percent change from baseline in HDL-C levels at 24 weeks (subgroup analysis).


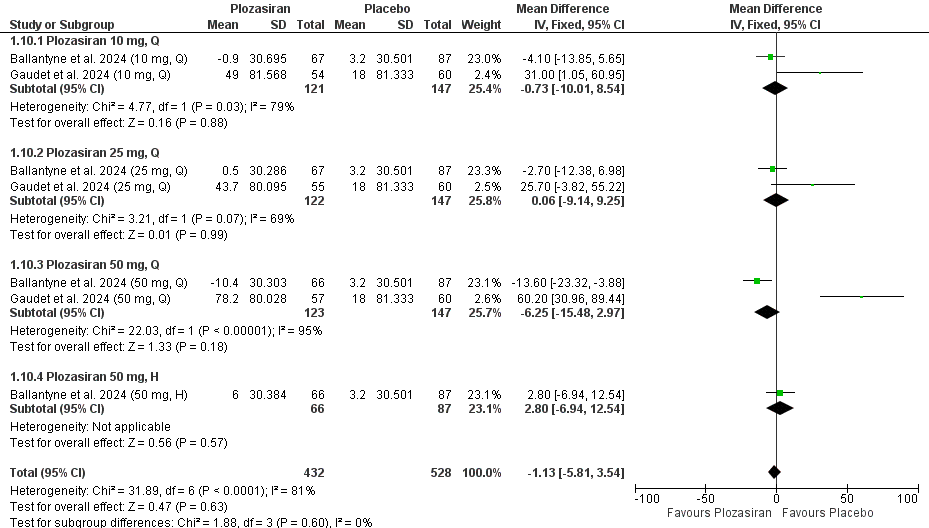


Supplementary Figure 21: Forrest plot demonstrating percent change from baseline in LDL-C levels at 24 weeks (subgroup analysis).


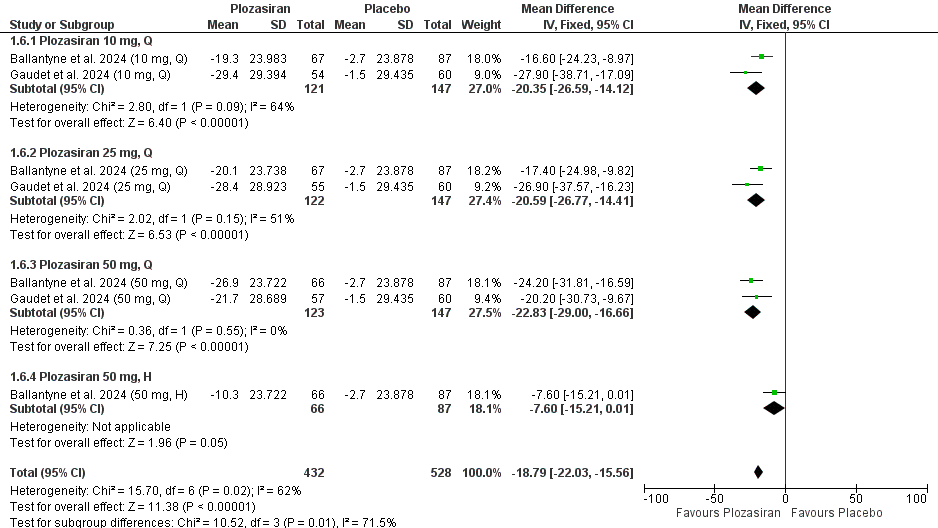


Supplementary Figure 22: Forrest plot demonstrating percent change from baseline in Non-HDL cholesterol levels at 24 weeks (subgroup analysis).


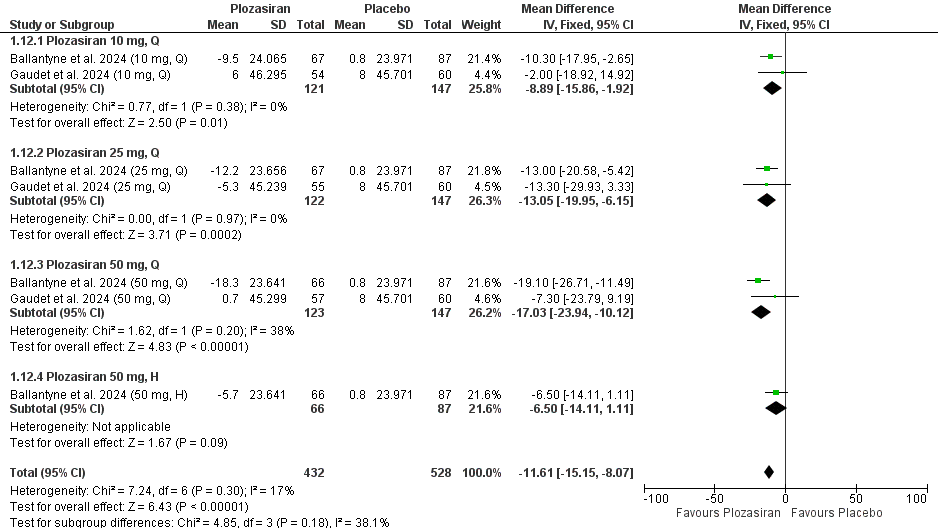


Supplementary Figure 23: Forrest plot demonstrating percent change from baseline in ApoB levels at 24 weeks (subgroup analysis).


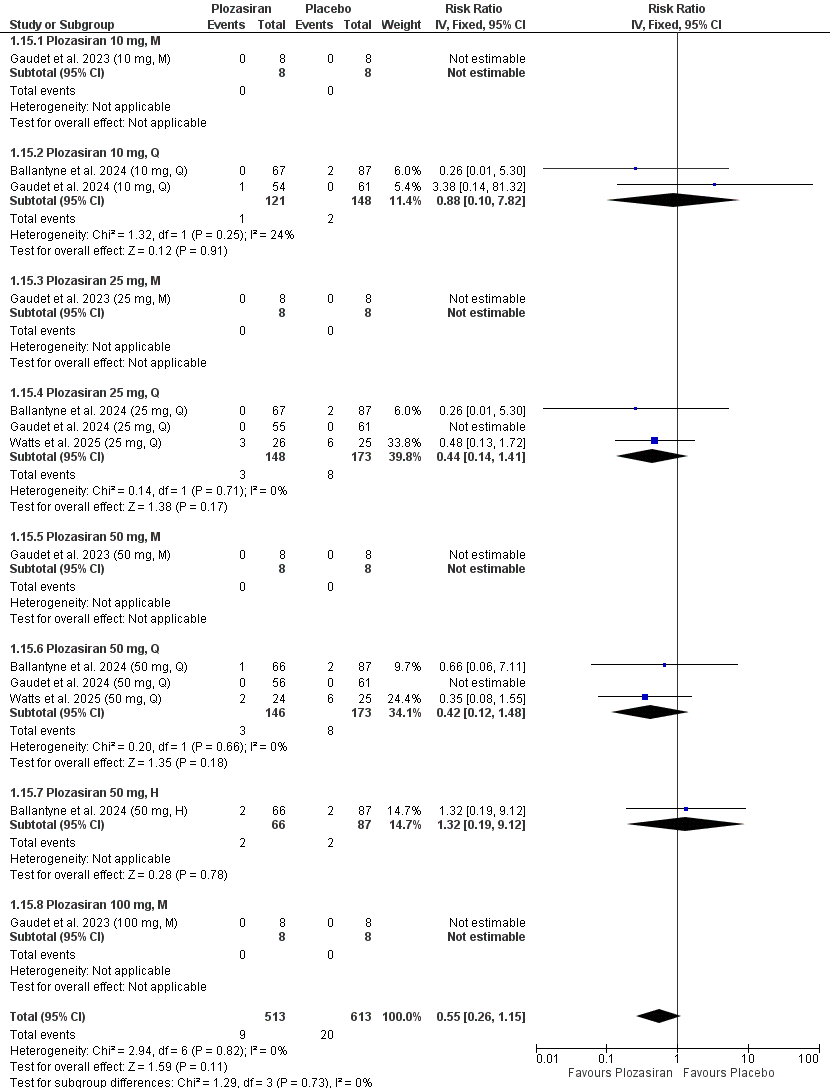


Supplementary Figure 24: Forrest plot demonstrating adverse events leading to discontinuation of the drug at the end of the study (subgroup analysis).


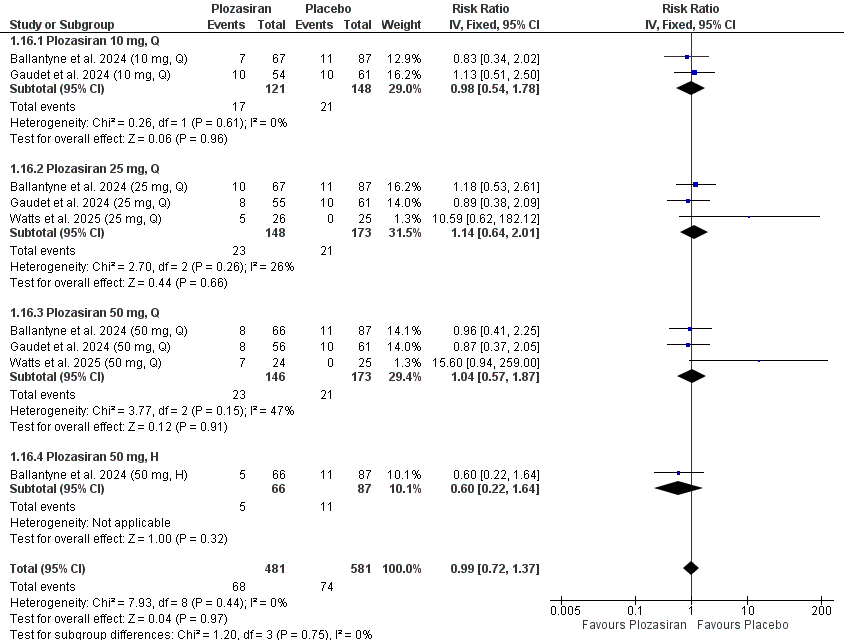


Supplementary Figure 25: Forrest plot demonstrating Covid-19 at the end of the study (subgroup analysis).


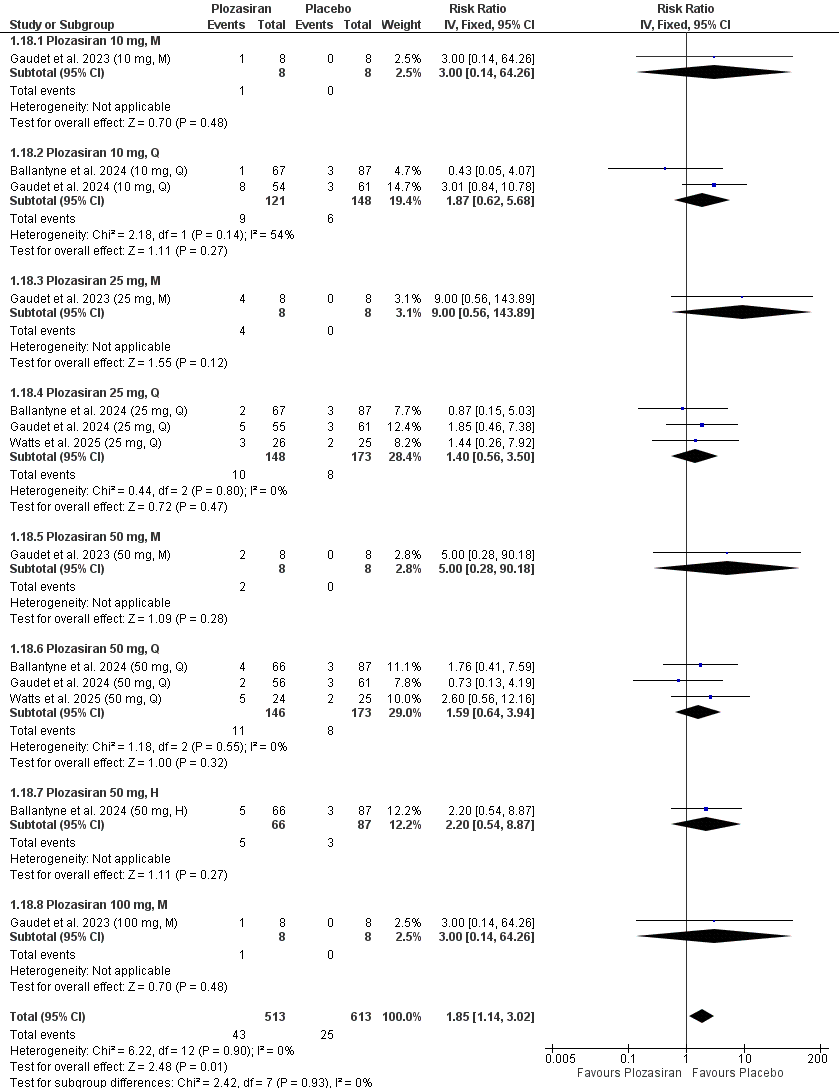


Supplementary Figure 26: Forrest plot demonstrating headache at the end of the study (subgroup analysis).


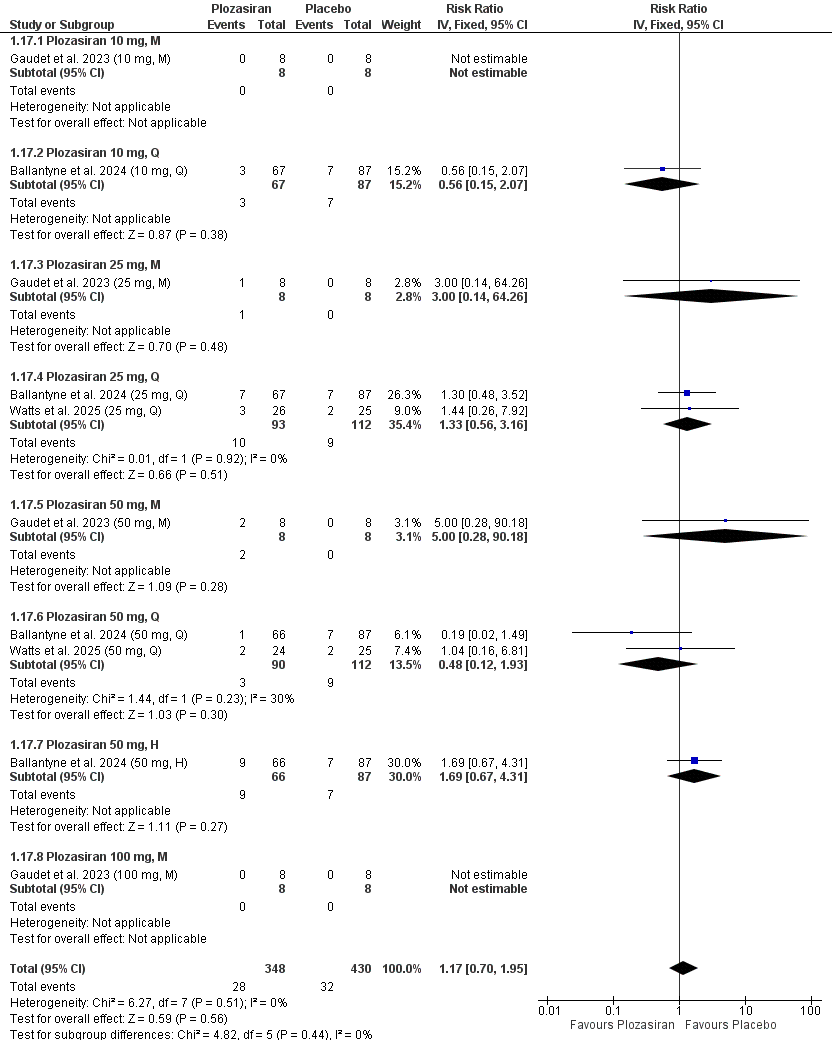


Supplementary Figure 27: Forrest plot demonstrating URTI at the end of the study (subgroup analysis).


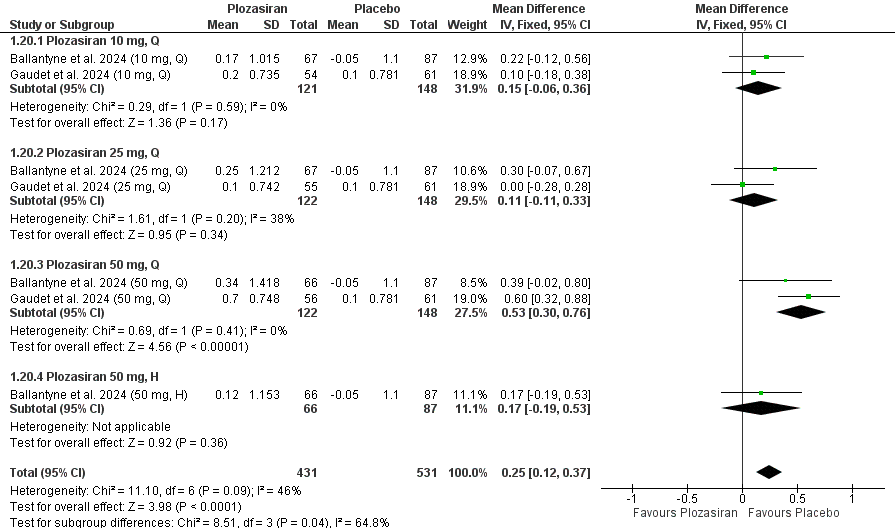


Supplementary Figure 28A: Forrest plot demonstrating absolute change from baseline in HbA1C levels at 24 weeks (subgroup analysis).


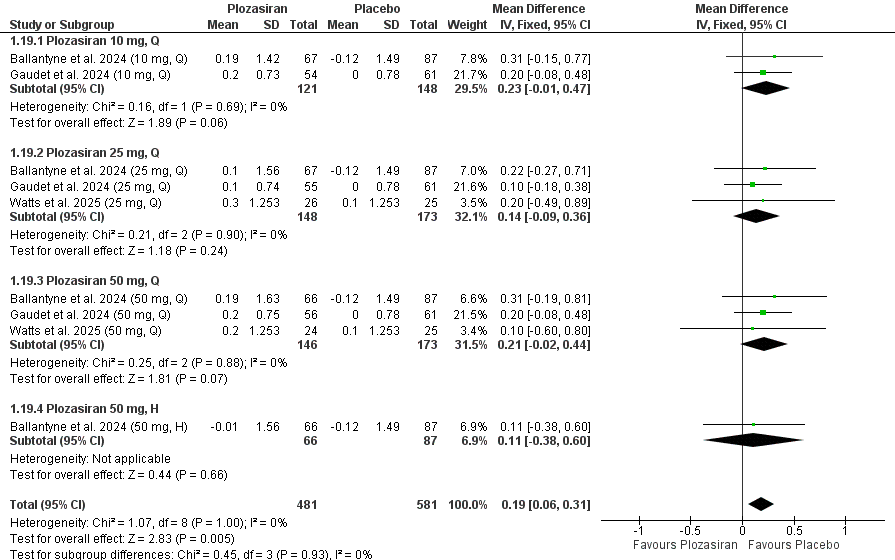


Supplementary Figure 28B: Forrest plot demonstrating absolute change from baseline in HbA1C levels at the end of the study (subgroup analysis).


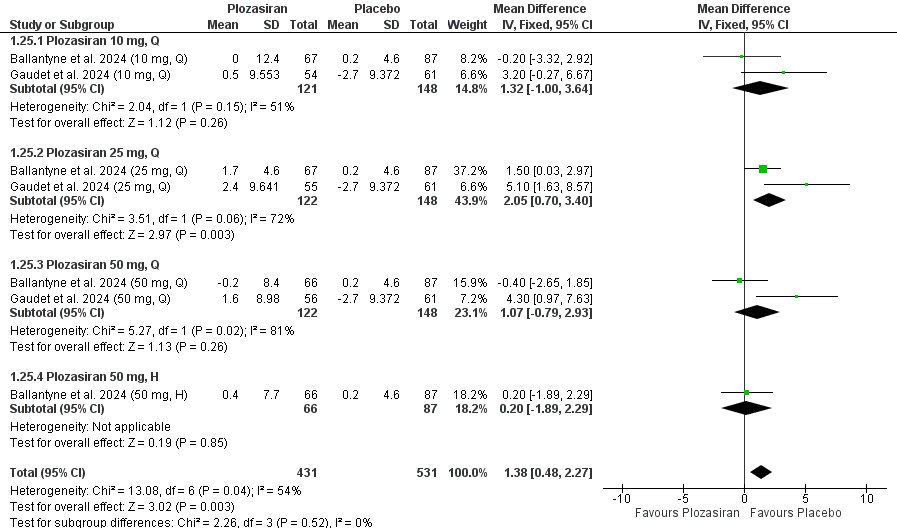


Supplementary Figure 29A: Forrest plot demonstrating absolute change from baseline in AST levels at 24 weeks (subgroup analysis).


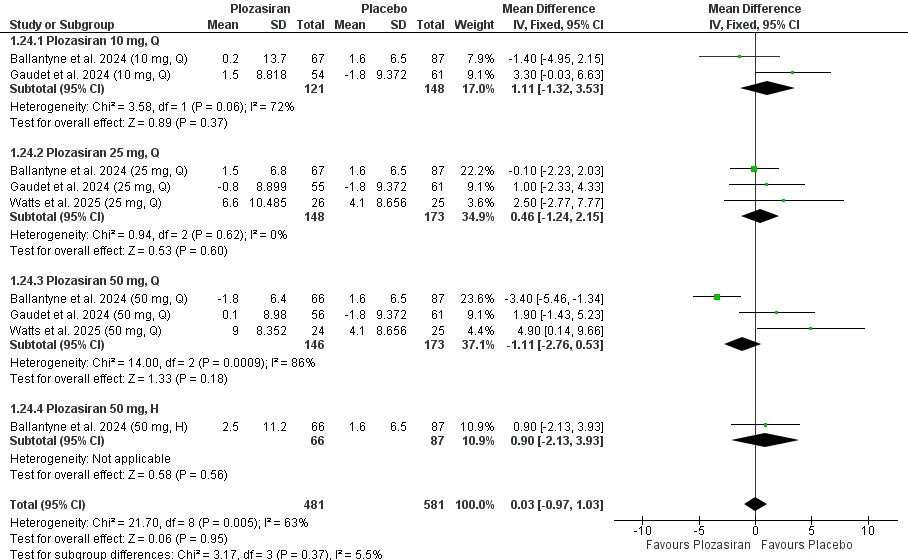


Supplementary Figure 29B: Forrest plot demonstrating absolute change from baseline in AST levels at the end of the study (subgroup analysis).


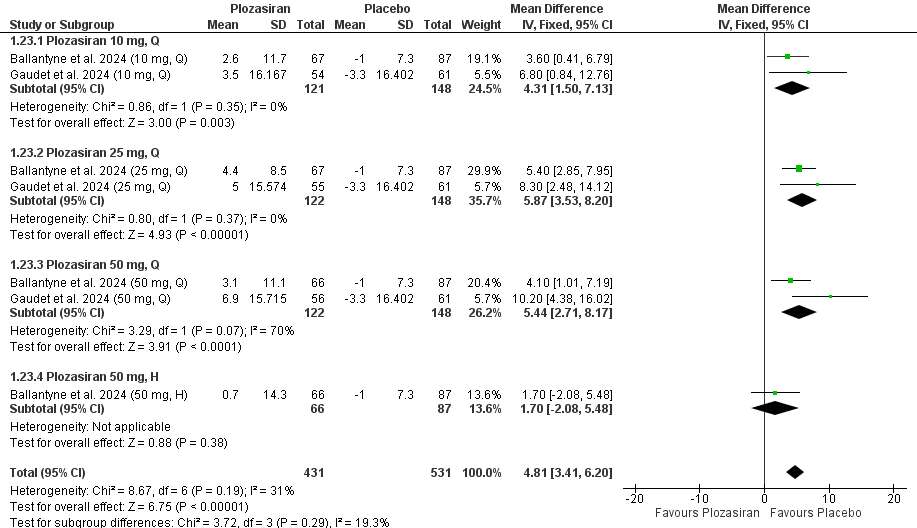


Supplementary Figure 30A: Forrest plot demonstrating absolute change from baseline in ALT levels at 24 weeks (subgroup analysis).


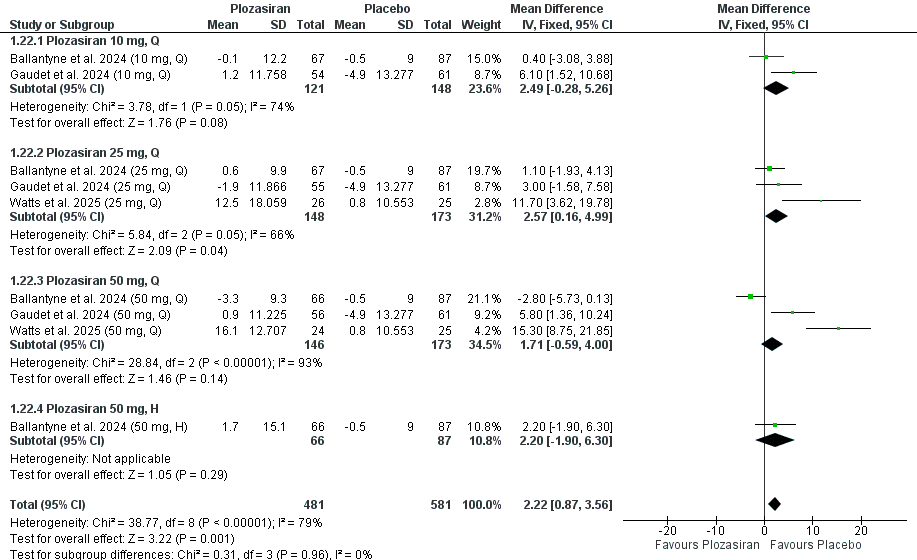


Supplementary Figure 30B: Forrest plot demonstrating absolute change from baseline in ALT levels at the end of the study (subgroup analysis).


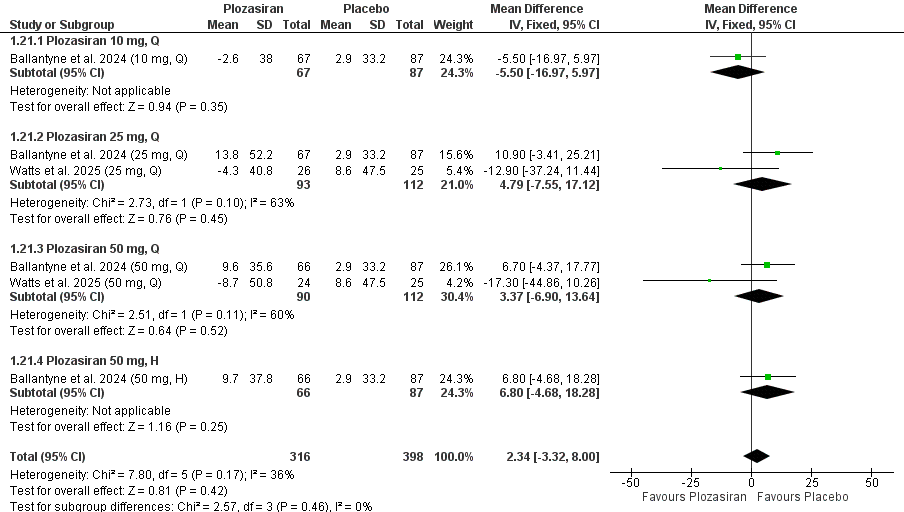


Supplementary Figure 31: Forrest plot demonstrating absolute change from baseline in platelets count at the end of the study (subgroup analysis).
